# Time-varying graph representation learning via higher-order skip-gram with negative sampling

**DOI:** 10.1140/epjds/s13688-022-00344-8

**Published:** 2022-05-28

**Authors:** Simone Piaggesi, André Panisson

**Affiliations:** 1 Alma Mater Studiorum University of Bologna, Bologna, Italy; 2 ISI Foundation, Turin, Italy; 3 CENTAI, Turin, Italy

**Keywords:** Representation learning, Time-varying graphs, Spreading processes, Temporal link prediction

## Abstract

Representation learning models for graphs are a successful family of techniques that project nodes into feature spaces that can be exploited by other machine learning algorithms. Since many real-world networks are inherently dynamic, with interactions among nodes changing over time, these techniques can be defined both for static and for time-varying graphs. Here, we show how the skip-gram embedding approach can be generalized to perform implicit tensor factorization on different tensor representations of time-varying graphs. We show that higher-order skip-gram with negative sampling (HOSGNS) is able to disentangle the role of nodes and time, with a small fraction of the number of parameters needed by other approaches. We empirically evaluate our approach using time-resolved face-to-face proximity data, showing that the learned representations outperform state-of-the-art methods when used to solve downstream tasks such as network reconstruction. Good performance on predicting the outcome of dynamical processes such as disease spreading shows the potential of this method to estimate contagion risk, providing early risk awareness based on contact tracing data.

**Supplementary Information:**

The online version contains supplementary material available at 10.1140/epjds/s13688-022-00344-8.

## Introduction

A great variety of natural and artificial systems can be represented as networks of elementary structural entities coupled by relations between them. The abstraction of such systems as networks helps us understand, predict and optimize their behaviour [1, 2]. In this sense, node and graph embeddings have been established as standard feature representations in many learning tasks [3, 4]. Node embedding methods map nodes into low-dimensional vectors that can be used to solve downstream tasks such as edge prediction, network reconstruction and node classification.

Node embeddings have proven successful in achieving low-dimensional encoding of static network structures, but many real-world networks are inherently dynamic [5, 6]. Time-resolved networks are also the support of important dynamical processes, such as epidemic or rumor spreading, cascading failures, consensus formation, etc. [7]. Time-resolved node embeddings have been shown to yield improved performance for predicting the outcome of dynamical processes over networks, such as information diffusion and disease spreading [8], providing estimation of infection and contagion risk when used with contact tracing data.

Since we expect having more data on proximity networks being used for contact tracing and as proxies for epidemic risk [9], learning meaningful representations of time-resolved proximity networks can be of extreme importance when facing events such as epidemic outbreaks [10, 11]. The manual and automatic collection of time-resolved proximity graphs for contact tracing purposes presents an opportunity for quick identification of possible infection clusters and infection chains. Even before the COVID-19 pandemic, the use of wearable proximity sensors for collecting time-resolved proximity networks has been largely discussed in the literature and many approaches have been used to describe patterns of activity and community structure, and to study spreading patterns of infectious diseases [12–14].

Here we propose a representation learning model that performs implicit tensor factorization on different higher-order representations of time-varying graphs. The main contributions are as follows:

- Given that the skip-gram embedding approach implicitly performs a factorization of the shifted *pointwise mutual information* matrix (PMI) [15], we generalize it to perform implicit factorization of a shifted PMI tensor. We then define the steps to achieve this factorization using higher-order skip-gram with negative sampling (HOSGNS) optimization.
- We show how to apply 3rd-order and 4th-order SGNS on different higher-order representations of time-varying graphs.
- We show that time-varying graph representations learned via HOSGNS outperform state-of-the-art methods when used to solve downstream tasks, even using a fraction of the number of embedding parameters.

We report the results of learning embeddings on empirical time-resolved face-to-face proximity data and using such representations as predictors for solving three different tasks: predicting the outcomes of a SIR spreading process over the time-varying graph, network reconstruction and link prediction. We compare these results with state-of-the art methods for time-varying graph representation learning.

## Preliminaries and related work

### Skip-gram representation learning

The skip-gram model was designed to compute word embeddings in word2vec [16], and afterwards extended to graph node embeddings [17–19]. Levy and Goldberg [15] established the relation between skip-gram trained with negative sampling (SGNS) and traditional matrix decomposition methods [20, 21], showing the equivalence of SGNS optimization to factorizing a shifted PMI matrix [22].

Starting from a textual corpus of words $w_{1},w_{2},\dots , w_{m}$ from a vocabulary $\mathcal{V}$, it assigns to each word $w_{s}$ a context corresponding to words surrounding $w_{s}$ in a window of size *T*, i.e. the multi-set $c_{T}(w_{s}) = \{w_{s-T},\dots ,w_{s-1},w_{s+1},\dots ,w_{s+T}\}$. Training samples $\mathcal{D} = \{(i,j): i \in \mathcal{W}, j \in \mathcal{C},~j \in c_{T}(i) \}$ are built by collecting all the observed word-context pairs, where $\mathcal{W}$ and $\mathcal{C}$ are the vocabularies of words and contexts respectively (usually $\mathcal{W} = \mathcal{C} = \mathcal{V}$). Here we denote as $\#(i,j)$ the number of times $(i,j)$ appears in $\mathcal{D}$. Similarly we use $\#i = \sum_{j}\#(i,j)$ and $\#j = \sum_{i}\#(i,j)$ as the number of times each word occurs in $\mathcal{D}$, with relative frequencies $P_{\mathcal{D}}(i,j)= \frac{\#(i,j)}{|\mathcal{D}|}$, $P_{\mathcal{D}}(i)= \frac{\#i}{|\mathcal{D}|}$ and $P_{\mathcal{D}}(j)= \frac{\#j}{|\mathcal{D}|}$.

SGNS computes *d*-dimensional representations for words and contexts in two matrices $\mathbf{W} \in \mathbb{R}^{|\mathcal{W}| \times d}$ and $\mathbf{C} \in \mathbb{R}^{|\mathcal{C}| \times d}$, performing a binary classification task in which pairs $(i,j) \in \mathcal{D}$ are positive examples and pairs $(i,j_{\mathcal{N}})$ with randomly sampled contexts are negative examples. The probability of the positive class is parametrized as the sigmoid ($\sigma (x) = (1+e^{-x})^{-1}$) of the inner product of embedding vectors:

1 $$ P\bigl[(i,j) \in \mathcal{D} \mid \mathbf{w}_{i}, \mathbf{c}_{j} \bigr] = \sigma ( \mathbf{w}_{i}\cdot \mathbf{c}_{j}) $$

and each word-context pair $(i,j)$ contributes to the loss as follows:

2 $$ \ell (i,j) = \log \sigma (\mathbf{w}_{i}\cdot \mathbf{c}_{j}) + \kappa \cdot \underset{j_{\mathcal{N}} \sim P_{\mathcal{N}}}{\mathbb{E}}\bigl[ \log \sigma (-\mathbf{w}_{i} \cdot \mathbf{c}_{j_{\mathcal{N}}})\bigr], $$

where the second expression uses the symmetry property $\sigma (-x) = 1-\sigma (x)$ inside the expected value and *κ* is the number of negative examples, sampled according to the empirical distribution of contexts $P_{\mathcal{N}}(j) = P_{\mathcal{D}}(j)$.

Following results found in [15], the sum of all $\ell (i,j)$ weighted with the probability that each pair $(i,j)$ appears in $\mathcal{D}$ gives the SGNS objective function:

3 $$ \mathcal{L}^{\mathrm{SGNS}} = - \sum _{i=1}^{ |\mathcal{W}|}\sum_{j=1}^{| \mathcal{C}|} \bigl[P_{\mathcal{D}}(i, j) \log \sigma (\mathbf{w}_{i} \cdot \mathbf{c}_{j}) + \kappa \, P_{\mathcal{N}}(i, j) \log \sigma (- \mathbf{w}_{i}\cdot \mathbf{c}_{j}) \bigr], $$

where $P_{\mathcal{N}}(i, j) = P_{\mathcal{D}}(i) \cdot P_{\mathcal{D}}(j)$ is the probability of $(i,j)$ under assumption of statistical independence.

Levy and Goldberg [15] demonstrated that, when *d* is sufficiently high, optimal SGNS embedding matrices satisfy these relations:

4 $$ \bigl(\mathbf{W}\mathbf{C}^{\mathrm{T}}\bigr)_{ij} \approx \log \biggl( \frac{P_{\mathcal{D}}(i, j)}{\kappa ~P_{\mathcal{N}}(i, j)} \biggr) = \operatorname{PMI}(i,j) - \log ( \kappa ) $$

which tell us that SGNS optimization is equivalent to a rank-*d* matrix decomposition of the word-context pointwise mutual information (PMI) matrix shifted by a constant, i.e. the number of negative samples. Here in this work, we refer to the shifted PMI matrix also as $\operatorname{SPMI}_{\kappa} = \operatorname{PMI} -\log \kappa $. This equivalence was later retrieved from diverse assumptions [23–27], and exploited to compute closed form expressions approximated in different graph embedding models [28].

### Random walk based graph embeddings

Given an undirected, weighted and connected graph $\mathcal{G = (V,E)}$ with nodes $i,j \in \mathcal{V}$, edges $(i,j) \in \mathcal{E}$ and adjacency matrix **A**, graph embedding methods are unsupervised models designed to map nodes into dense *d*-dimensional representations ($d \ll |\mathcal{V}|$) [29]. A well known family of approaches based on the skip-gram model consists in sampling random walks from the graph and processing node sequences as textual sentences. In DeepWalk [17] and node2vec [19], the skip-gram model is used to obtain node embeddings from co-occurrences in random walk realizations. Although the original implementation of DeepWalk uses hierarchical softmax to compute embeddings, we will refer to the SGNS formulation given by [28].

Since SGNS can be interpreted as a factorization of the word-context PMI matrix [15], the asymptotic form of the PMI matrix implicitly decomposed in DeepWalk can be derived [28]. Given the 1-step transition matrix $\mathbf{P} = \mathbf{D}^{-1}\mathbf{A}$, where $\mathbf{D} = \operatorname{diag}(d_{1}, \dots , d_{|\mathcal{V}|})$ and $d_{i} = \sum_{j \in \mathcal{V}}\mathbf{A}_{ij}$ is the (weighted) node degree, the expected PMI for a node-context pair $(i,j)$ occurring in a *T*-sized window is:

5 $$ \operatorname{PMI}^{\mathrm{DW}}(i,j) = \log \biggl( \frac{P_{\mathcal{D}}(i, j)}{P_{\mathcal{N}}(i,j)} \biggr) = \log \biggl(\frac{ \overbrace{\textstyle \frac{1}{2T}\sum_{r=1}^{T} [ \frac{d_{i}}{\operatorname{vol}(\mathcal{G})}(\mathbf{P}^{r})_{ij} + \frac{d_{j}}{\operatorname{vol}(\mathcal{G})} (\mathbf{P}^{r})_{ji} ] }^{(a)} }{ \frac{d_{i}}{\operatorname{vol}(\mathcal{G})}\cdot \frac{d_{j}}{\operatorname{vol}(\mathcal{G})} }\biggr), $$

where $\operatorname{vol}(\mathcal{G}) = \sum_{i,j\in \mathcal{V}}\mathbf{A}_{ij}$. We will return to this equation in Sect. 3.2, where we use the expression in $(a)$ to build probability tensors from higher-order graph representations.

### Time-varying graphs and their algebraic representations

Time-varying graphs [5, 6] are defined as triples $\mathcal{H = (V,E,T)}$, i.e. collections of events $(i, j, k) \in \mathcal{E}$, representing undirected pairwise relations among nodes at discrete times ($i,j \in \mathcal{V}$, $k \in \mathcal{T}$). $\mathcal{H}$ can be seen as a temporal sequence of static graphs $\{\mathcal{G}^{(k)}\}_{k \in \mathcal{T}}$, each of those with adjacency matrix $\mathbf{A}^{(k)}$ such that $\mathbf{A}^{(k)}_{ij} = \omega (i,j,k) \in \mathbb{R}$ is the weight of the event $(i,j,k) \in \mathcal{E}$. We can concatenate the list of time-stamped snapshots $[\mathbf{A}^{(1)}, \dots , \mathbf{A}^{(|\mathcal{T}|)}]$ to obtain a single 3rd-order tensor $\boldsymbol{\mathcal{A}}^{\mathrm{stat}}(\mathcal{H}) \in \mathbb{R}^{| \mathcal{V}|\times |\mathcal{V}|\times |\mathcal{T}|}$ which characterize the evolution of the graph over time. This representation has been used to discover latent community structures of temporal graphs [13] and to perform temporal link prediction [30]. Indeed, beyond the above stacked graph representation, more exhaustive representations are possible. In particular, the multi-layer approach [31] allows to map the topology of a time-varying graph $\mathcal{H}$ into a static network $\mathcal{G_{\mathcal{H}}} = (\mathcal{V}_{\mathcal{H}}, \mathcal{E}_{ \mathcal{H}})$ (the *supra-adjacency* graph) such that vertices in $\mathcal{V}_{\mathcal{H}}$ correspond to node-time pairs $(i, k)\equiv i^{(k)} \in \mathcal{V} \times \mathcal{T}$ and edges in $\mathcal{E}_{\mathcal{H}}$ represent connections $(i^{(k)}, j^{(l)})$ among them. Since every link can be arranged in a quadruple $(i,j,k,l)$, the connectivity structure is associated to a 4th-order tensor $\boldsymbol{\mathcal{A}}^{\mathrm{dyn}}(\mathcal{H}) \in \mathbb{R}^{| \mathcal{V}|\times |\mathcal{V}|\times |\mathcal{T}|\times | \mathcal{T}|}$ that is equivalent, up to an opportune reshaping, to the adjacency matrix $\mathbf{A}(\mathcal{G}_{\mathcal{H}}) \in \mathbb{R}^{|\mathcal{V}|| \mathcal{T}|\times |\mathcal{V}||\mathcal{T}|}$ of $\mathcal{G}_{\mathcal{H}}$. Multi-layer representations for time-varying networks have been used to study time-dependent centrality measures [32] and properties of spreading processes [33].

In the same spirit that word2vec refers to the word pairs $(i,j)$ as (*word, context*), here we refer to the node pairs $(i,j)$ as (*node, context*), and the time pairs $(k,l)$ as (*time, context-time*).

### Time-varying graph representation learning

Given a time-varying graph $\mathcal{H} = (\mathcal{V}, \mathcal{E}, \mathcal{T})$, we define as temporal network embedding a model that learns from data, implicitly or explicitly, a mapping function:

6 $$ f : (v, t) \in \mathcal{V} \times \mathcal{T} \mapsto \mathbf{v}^{(t)} \in \mathbb{R}^{d} $$

which project time-stamped nodes into a latent low-rank vector space that encodes structural and temporal properties of the original evolving graph [34, 35]. Many existing methods learn node representations from sequences of static snapshots through incremental updates in a streaming scenario: deep autoencoders [36], SVD [37], skip-gram [38, 39] and random walk sampling [40–42]. Another class of models learn dynamic node representations by recurrent/attention mechanisms [43–46] or by imposing temporal stability among adjacent time intervals [47, 48]. DyANE [8] and weg2vec [49] project the dynamic graph structure into a static graph, in order to compute embeddings with word2vec. Closely related to these ones are [50] and [51], which learn node vectors according to time-respecting random walks or spreading trajectory paths. Moreover, [52] proposed an embedding framework for user-item temporal interactions, and [53] suggested a tensor-based convolutional architecture for dynamic graphs.

Methods that perform well for predicting outcomes of spreading processes make use of time-respecting supra-adjacency representations such as the one proposed by [33]. In these graph representations, a random walk corresponds to a temporal path in the original time-varying graph, enconding relevant information about the spreading process into its connectivity structure. The supra-adjacency representation $\mathcal{G}_{\mathcal{H}}$ that we refer in Sect. 3.2, also used in DyANE, with adjacency matrix $\mathbf{A}(\mathcal{G}_{\mathcal{H}})$, is defined by two rules:

1. For each event $(i,j,t_{0})$, if *i* is also active at time $t_{1} > t_{0}$ and in no other time-stamp between the two, we add a *cross-coupling* edge between supra-adjacency nodes $j^{(t_{0})}$ and $i^{(t_{1})}$. In addition, if the next interaction of *j* with other nodes happens at $t_{2}>t_{0}$, we add an edge between $i^{(t_{0})}$ and $j^{(t_{2})}$. The weights of such edges are set to $\omega (i,j,t_{0})$.
2. For every case as described above, we also add *self-coupling* edges $(i^{(t_{0})}, i^{(t_{1})})$ and $(j^{(t_{0})}, j^{(t_{2})})$, with weights set to 1.

**Figure 1:**
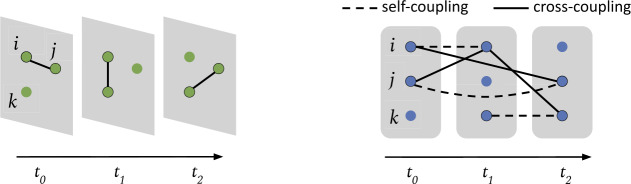
A time-varying graph $\mathcal{H}$ with three intervals (left) and its corresponding time-respecting supra-adjacency graph $\mathcal{G}_{\mathcal{H}}$ (right)

Figure 1 shows the differences between a time-varying graph and its time-aware supra-adjacency representation, according to the formulation described above. DyANE computes, given a node $i \in \mathcal{V}$, one vector representation for each time-stamped node $i^{(t)} \in \mathcal{V}^{(\mathcal{T})} = \{(i,t) \in \mathcal{V} \times \mathcal{T}: \exists (i,j,t) \in \mathcal{E}\}$ of this supra-adjacency representation. Similar models that learn time-resolved node representations require a quantity $\mathcal{O}(|\mathcal{V}|\cdot |\mathcal{T}|)$ of embedding parameters to represent the time-varying graph in the latent space. As we will see, compared with these methods, our approach requires a quantity $\mathcal{O}(|\mathcal{V}| + |\mathcal{T}|)$ of embedding parameters for disentangled node and time representations.

## Proposed method

Given a time-varying graph $\mathcal{H = (V, E, T)}$, we propose a representation learning method that learns disentangled representations for nodes and time slices. More formally, we learn a function:

7 $$ f^{\ast}: (v,t) \in \mathcal{V} \times \mathcal{T} \mapsto \mathbf{v}, \mathbf{t} \in \mathbb{R}^{d} $$

This embedding representation can then be reconciled with the definition in Eq. (6) by combining **v** and **t** in a single $\mathbf{v}^{(t)}$ representation using any combination function $c: (\mathbf{v},\mathbf{t}) \in \mathbb{R}^{d} \times \mathbb{R}^{d} \mapsto \mathbf{v}^{(t)} \in \mathbb{R}^{d}$. It follows that computing and combining distinct vector embeddings for nodes and time slices needs a quantity $\mathcal{O}(|\mathcal{V}|+|\mathcal{T}|)$ of learnable parameters, leading to a more efficient method to obtain time-aware node representations without requiring to learn a much bigger number $\mathcal{O}(|\mathcal{V}|\cdot |\mathcal{T}|)$ of learnable parameters.

The parameters of the embedding representation in Eq. (7) are learned through a higher-order generalization of skip-gram with negative sampling (HOSGNS), based on the existing skip-gram framework for node embeddings, as shown in Sect. 3.1. We show that this generalization allows to implicitly factorize higher-order relations that characterize tensor representations of time-varying graphs, in the same way that the classical SGNS decomposes dyadic relations associated to a static graph.

Similar approaches have been applied in NLP for dynamic word embeddings [54], and higher-order extensions of the skip-gram model have been proposed to learn context-dependent [55] and syntactic-aware [56] word representations. Also tensor factorization techniques have been applied to include the temporal dimension in recommender systems [57, 58], knowledge graphs [59, 60] and face-to-face contact networks [12, 13]. But this work is the first to merge SGNS with tensor factorization, and then apply it to learn time-varying graph embeddings. HOSGNS differs from existing temporal network embeddings based on skip-gram [38, 39], which are minor adaptations of standard SGNS to the dynamic setting. In fact, in Sect. 3.1 we show how the equations in the skip-gram framework can be completely rewritten to be naturally applied to inherently higher-order problems.

In the next sections, we first show the formal steps to the generalization of the skip-gram approach to higher-order data structures, and then we show how to apply HOSGNS on 3rd-order and 4th-order representations of time-varying graphs.

### SGNS for higher-order data structures

Here we address the problem of generalizing SGNS to learn embedding representations from higher-order co-occurrences. In Sect. 2.3 we described snapshot-based and multilayer-based representations of time-varying graphs, that can be seen as 3rd and 4th-order co-occurrence tensors; therefore in the remaining of this manuscript we focus on 3rd and 4th-order structures. In this section, we describe in detail the generalization of SGNS to the 3rd-order case. In Additional file 1 we discuss more in detail the derivation of the HOSGNS objective function to any *n*th-order representation.

We consider a set of training samples $\mathcal{D} = \{(i, j, k): i \in \mathcal{W}, j \in \mathcal{C}, k \in \mathcal{T}\}$ obtained by collecting co-occurrences among elements from three sets $\mathcal{W}$, $\mathcal{C}$ and $\mathcal{T}$. While SGNS is limited to pairs of node-context $(i, j)$, here $\mathcal{D}$ is constructed with three (or more) variables, e.g. sampling random walks over a higher-order data structure. We denote as $\#(i,j,k)$ the number of times the triple $(i,j,k)$ appears in $\mathcal{D}$. Similarly we use $\#i = \sum_{j,k}\#(i,j,k)$, $\#j = \sum_{i,k}\#(i,j,k)$ and $\#k = \sum_{i,j}\#(i,j,k)$ as the number of times each distinct element occurs in $\mathcal{D}$, with relative frequencies $P_{\mathcal{D}}(i,j,k)= \frac{\#(i,j,k)}{|\mathcal{D}|}$, $P_{\mathcal{D}}(i)= \frac{\#i}{|\mathcal{D}|}$, $P_{\mathcal{D}}(j)= \frac{\#j}{|\mathcal{D}|}$ and $P_{\mathcal{D}}(k)= \frac{\#k}{|\mathcal{D}|}$.

Optimization is performed as a binary classification task, where the objective is to discern occurrences actually coming from $\mathcal{D}$ from random occurrences. We define the likelihood for a single observation $(i,j,k)$ by applying a sigmoid ($\sigma (x) = (1+e^{-x})^{-1}$) to the higher-order inner product $[\!\![\cdot]\!\!]$ of corresponding *d*-dimensional representations:

8 $$ P\bigl[(i,j,k) \in \mathcal{D} \mid \mathbf{w}_{i}, \mathbf{c}_{j}, \mathbf{t}_{k}\bigr] = \sigma \bigl( [\!\![\mathbf{w}_{i}, \mathbf{c}_{j}, \mathbf{t}_{k}]\!\!]\bigr) \equiv \sigma \Bigl(\sum \nolimits _{r=1}^{d} \mathbf{W}_{ir} \mathbf{C}_{jr} \mathbf{T}_{kr} \Bigr), $$

where embedding vectors $\mathbf{w}_{i},\mathbf{c}_{j}, \mathbf{t}_{k} \in \mathbb{R}^{d}$ are respectively rows of $\mathbf{W} \in \mathbb{R}^{|\mathcal{W}| \times d}$, $\mathbf{C} \in \mathbb{R}^{|\mathcal{C}| \times d}$ and $\mathbf{T} \in \mathbb{R}^{|\mathcal{T}| \times d}$. In the 4th-order case we will also have a fourth embedding matrix $\mathbf{S} \in \mathbb{R}^{|\mathcal{S}| \times d}$ related to a fourth set $\mathcal{S}$. For negative sampling we fix an observed $(i,j,k) \in \mathcal{D}$ and independently sample $j_{\mathcal{N}}$ and $k_{\mathcal{N}}$ to generate *κ* negative examples $(i,j_{\mathcal{N}},k_{\mathcal{N}})$. In this way, for a single occurrence $(i,j,k) \in \mathcal{D}$, the expected contribution to the loss is:

9 $$ \ell (i,j,k) = \log \sigma \bigl([\!\![\mathbf{w}_{i}, \mathbf{c}_{j}, \mathbf{t}_{k} ]\!\!]\bigr) + \kappa \cdot \underset{j_{\mathcal{N}}, k_{\mathcal{N}} \sim P_{\mathcal{N}}}{\mathbb{E}} \bigl[\log \sigma \bigl(-[\!\![\mathbf{w}_{i}, \mathbf{c}_{j_{\mathcal{N}}}, \mathbf{t}_{k_{\mathcal{N}}}]\!\!]\bigr) \bigr], $$

where the noise distribution is the product of independent marginal probabilities $P_{\mathcal{N}}(j, k)= P_{\mathcal{D}}(j) \cdot P_{\mathcal{D}}(k)$. Thus the global objective is the sum of all the quantities of Eq. (9) weighted with the corresponding relative frequency $P_{\mathcal{D}}(i,j,k)$. The full loss function can be expressed as:

10 $$ \mathcal{L} = - \sum_{i=1}^{ |\mathcal{W}|} \sum_{j=1}^{|\mathcal{C}|} \sum _{k=1}^{ |\mathcal{T}|} \bigl[ P_{\mathcal{D}}(i,j,k) \log \sigma \bigl([\!\![\mathbf{w}_{i}, \mathbf{c}_{j}, \mathbf{t}_{k}]\!\!]\bigr) + \kappa \ P_{\mathcal{N}}(i,j,k) \log \sigma \bigl(-[\!\![\mathbf{w}_{i}, \mathbf{c}_{j}, \mathbf{t}_{k}]\!\!]\bigr) \bigr]. $$

In Additional file 1 we show the formal steps to obtain Eq. (10) for the *n*th-order case and that it can be optimized with respect to the embedding parameters, satisfying the low-rank tensor approximation of the multivariate shifted PMI tensor into factor matrices **W**, **C**, **T**:

11 $$ \sum \nolimits _{r=1}^{d} \mathbf{W}_{ir} \mathbf{C}_{jr} \mathbf{T}_{kr} \approx \log \biggl( \frac{P_{\mathcal{D}}(i,j,k)}{P_{\mathcal{N}}(i,j,k)} \biggr) - \log \kappa \equiv \operatorname{SPMI}_{\kappa}(i,j,k). $$

Equation (11), like the analogous derived in Levy and Goldberg [15] in Eq. (4), assumes full rank embedding matrices with $d \approx R = \operatorname{rank}(\operatorname{SPMI}_{\kappa})$. For the case when $d \ll R$, a comprehensive theoretical analysis is missing, although recent works propose the feasibility of exact low-dimensional factorizations of real-world static networks [61, 62]. Nevertheless, in Additional file 1, we include an empirical analysis of the effectiveness of HOSGNS for low-rank factorization of time-varying graph representations.

### Time-varying graph embedding via HOSGNS

While a static graph $\mathcal{G = (V,E)}$ is uniquely represented by an adjacency matrix $\mathbf{A}(\mathcal{G}) \in \mathbb{R}^{|\mathcal{V}|\times | \mathcal{V}|}$, a time-varying graph $\mathcal{H = (V,E,T)}$ admits diverse possible higher-order adjacency relations (Sect. 2.3). Starting from these higher-order relations, we can either use them directly or use random walk realizations to build a dataset of higher-order co-occurrences. In the same spirit that random walk realizations lead to dyadic co-occurrences used to learn embeddings in SGNS, we use higher-order co-occurrences to learn embeddings via HOSGNS.

As discussed in Sect. 3.1, the statistics of higher-order relations can be summarized in multivariate PMI tensors, which derive from co-occurrence probabilities among elements. Once such PMI tensors are constructed, we can again factorize them via HOSGNS. To show the versatility of this approach, we choose probability tensors derived from two different types of higher-order relations:

1. A 3rd-order tensor $\boldsymbol{\mathcal{P}}^{(\mathrm{stat})}(\mathcal{H}) \in \mathbb{R}^{| \mathcal{V}|\times |\mathcal{V}|\times |\mathcal{T}|}$ which gather relative frequencies of nodes occurrences in temporal edges:
  12 $$ \bigl(\boldsymbol{\mathcal{P}}^{(\mathrm{stat})} \bigr)_{ijk} = \frac{\omega (i,j,k)}{\operatorname{vol}(\mathcal{H})}, $$
  where $\operatorname{vol}(\mathcal{H}) = \sum_{i,j,k}\omega (i,j,k)$ is the total weight of interactions occurring in $\mathcal{H}$. These probabilities are associated to the snapshot sequence representation $\boldsymbol{\mathcal{A}}^{\mathrm{stat}}(\mathcal{H}) = [\mathbf{A}^{(1)}, \dots , \mathbf{A}^{(|\mathcal{T}|)}]$ and contain information about the topological structure of $\mathcal{H}$.
2. A 4th-order tensor $\boldsymbol{\mathcal{P}}^{(\mathrm{dyn})}(\mathcal{H}) \in \mathbb{R}^{| \mathcal{V}|\times |\mathcal{V}|\times |\mathcal{T}|\times | \mathcal{T}|}$, which gather occurrence probabilities of time-stamped nodes over random walks of the supra-adjacency graph $\mathcal{G}_{\mathcal{H}}$ (as used in DyANE). Using the numerator of Eq. (5), with supra-adjacency indices $i^{(k)}$ and $j^{(l)}$ instead of usual indices *i* and *j*, tensor entries are given by:
  13 $$ \bigl(\boldsymbol{\mathcal{P}}^{(\mathrm{dyn})} \bigr)_{ijkl} = \frac{1}{2T}\sum_{r=1}^{T} \biggl[ \frac{d_{i^{(k)}}}{\operatorname{vol}(\mathcal{G}_{\mathcal{H}})}\bigl( \mathbf{P}^{r} \bigr)_{i^{(k)},j^{(l)}} + \frac{d_{j^{(l)}}}{\operatorname{vol}(\mathcal{G}_{\mathcal{H}})} \bigl( \mathbf{P}^{r} \bigr)_{j^{(l)},i^{(k)}} \biggr]. $$
  These probabilities encode causal dependencies among temporal nodes and are correlated with dynamical properties of spreading processes. Notice that the computation of $\boldsymbol{\mathcal{P}}^{(\mathrm{dyn})}(\mathcal{H})$ requires an undirected supra-adjacency graph, while in DyANE is directed.

We also combined the two representations in a single tensor that is the average of $\boldsymbol{\mathcal{P}}^{(\mathrm{stat})}$ and $\boldsymbol{\mathcal{P}}^{(dyn)}$

14 $$ \bigl(\boldsymbol{\mathcal{P}}^{(\mathrm{stat}|\mathrm{dyn})}\bigr)_{ijkl} = \frac{1}{2} \bigl[\bigl( \boldsymbol{\mathcal{P}}^{(\mathrm{stat})} \bigr)_{ijk} \delta _{kl} + \bigl( \boldsymbol{ \mathcal{P}}^{(\mathrm{dyn})}\bigr)_{ijkl} \bigr], $$

where $\delta_{kl}=1[k=l]$ is the Kronecker delta.

**Figure 2:**
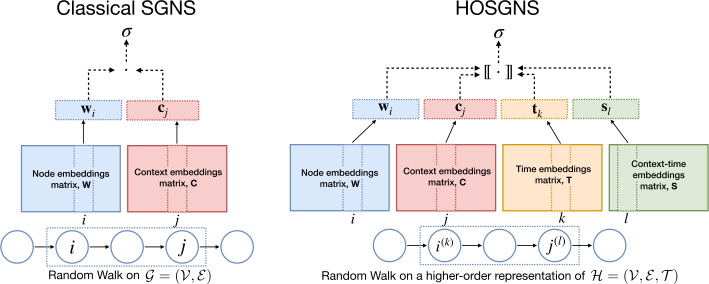
Representation of SGNS and HOSGNS with embedding matrices and operations on embedding vectors. Starting from a random walk realization on a static graph $\mathcal{G=(V,E)}$, SGNS takes as input nodes *i* and *j* within a context window of size *T*, and maximizes $\sigma (\mathbf{w}_{i} \cdot \mathbf{c}_{j})$. HOSGNS starts from a random walk realization on a higher-order representation of time-varying graph $\mathcal{H=(V,E,T)}$, takes as input nodes $i^{(k)}$ (node *i* at time *k*) and $j^{(l)}$ (node *j* at time *l*) within a context window of size *T* and maximizes $\sigma ([\!\![\mathbf{w}_{i}, \mathbf{c}_{j}, \mathbf{t}_{k}, \mathbf{s}_{l}]\!\!])$. In both cases, for each input sample, we fix *i* and draw *κ* combinations of *j* or *j*, *k*, *l* from a noise distribution, and we maximize $\sigma (-\mathbf{w}_{i} \cdot \mathbf{c}_{j})$ (SGNS) or $\sigma (-[\!\![\mathbf{w}_{i}, \mathbf{c}_{j}, \mathbf{t}_{k}, \mathbf{s}_{l}]\!\!])$ (HOSGNS) with their corresponding embedding vectors (negative sampling)

Figure 2 summarizes the differences between graph embedding via classical SGNS and time-varying graph embedding via HOSGNS. Here, indices $(i,j,k,l)$ correspond to *(node, context, time, context-time)* in a 4th-order tensor representation of $\mathcal{H}$.

The above tensors gather empirical probabilities $P_{\mathcal{D}}(i,j,k\dots )$ corresponding to positive examples of observable higher-order relations. The probabilities of negative examples $P_{\mathcal{N}}(i,j,k\dots )$ can be obtained as the product of marginal distributions $P_{\mathcal{D}}(i)$, $P_{\mathcal{D}}(j)$, $P_{\mathcal{D}}(k)\dots $ Objective functions like Eq. (10) can be computed with a sampling strategy: picking positive tuples according to the data distribution $P_{\mathcal{D}}$ and negative ones according to independent sampling $P_{\mathcal{N}}$, HOSGNS objective can be optimized through the following weighted cross entropy loss:

15 $$ \mathcal{L}^{(\mathrm{bce})} = -\frac{1}{B} \Biggl[ \sum_{(ijk\dots ) \sim P_{\mathcal{D}}}^{B} \log \sigma \bigl( [\!\![\mathbf{w}_{i}, \mathbf{c}_{j}, \mathbf{t}_{k},\dots]\!\!]\bigr) + \kappa \cdot \sum _{(ijk \dots ) \sim P_{\mathcal{N}}}^{B} \log \sigma \bigl(-[\!\![\mathbf{w}_{i}, \mathbf{c}_{j}, \mathbf{t}_{k}, \dots]\!\!]\bigr) \Biggr], $$

where *B* is the number of the samples drawn in a training step and *κ* is the negative sampling constant. We additionally apply the *warm-up* steps explained in Additional file 1 to speed-up the main training stage.

## Experiments

For the experiments we use time-varying graphs collected by the SocioPatterns collaboration (http://www.sociopatterns.org) using wearable proximity sensors that sense the face-to-face proximity relations of individuals wearing them. After training the proposed models (HOSGNS applied to $\boldsymbol{\mathcal{P}}^{(\mathrm{stat})}$, $\boldsymbol{\mathcal{P}}^{(\mathrm{dyn})}$ or $\boldsymbol{\mathcal{P}}^{(\mathrm{stat}|\mathrm{dyn})}$) on each dataset, embedding matrices $\mathbf{W, C, T}$ (and **S** except for $\boldsymbol{\mathcal{P}}^{(\mathrm{stat})}$) are mapped to embedding vectors $\mathbf{w}_{i}$, $\mathbf{c}_{j}$, $\mathbf{t}_{k}$ (and $\mathbf{s}_{l}$) where $i,j \in \mathcal{V}$ and $k,l \in \mathcal{T}$. In Sect. 4.2, we use the learned representations to solve different downstream tasks: *node classification*, *temporal event reconstruction* and *missing event prediction*. Finally, in Sect. 4.4 we show the visualization of the two-dimensional projections of the embeddings for one of the chosen empirical datasets.

### Experimental setup

#### Datasets

We performed experiments with both empirical and synthetic datasets describing face-to-face proximity of individuals. We used publicly available empirical contact data collected by the SocioPatterns collaboration [63], with a temporal resolution of 20 seconds, in a variety of contexts: in a school (“LyonSchool”), a conference (“SFHH”), a hospital (“LH10”), a highschool (“Thiers13”), and in offices (“InVS15”) [64]. This is currently the largest collection of open datasets sensing proximity in the same range and temporal resolution used by modern contact tracing systems. In addition, we used social interactions data generated by the agent-based-model OpenABM-Covid19 [65] to simulate an outbreak of COVID-19 in a urban setting.

We built a time-varying graph from each dataset, and for the empirical data we performed aggregation on 600 seconds time windows, neglecting those snapshots without registered interactions at that time scale. The weight of the link $(i,j,k)$ is the number of events recorded between nodes $(i,j)$ in a certain aggregated window *k*. For synthetic data we maintained the original temporal resolution and we set links weights to 1. Table 1 shows statistics for each dataset.

**Table 1:** Summary statistics about empirical and synthetic time-varying graph data. In order: number of single nodes $|\mathcal{V}|$, number of steps $|\mathcal{T}|$, number of events $|\mathcal{E}|$, number of active nodes $|\mathcal{V}^{(\mathcal{T})}|$, average weight of events $\frac{1}{|\mathcal{E}|}\sum_{e \in \mathcal{E}} \omega (e)$, nodes density $\frac{|\mathcal{V}^{(\mathcal{T})}|}{|\mathcal{V}||\mathcal{T}|}$ and links density $\frac{2|\mathcal{E}|}{|\mathcal{V}|(|\mathcal{V}|-1)|\mathcal{T}|}$

Dataset	$|\mathcal{V}|$	$|\mathcal{T}|$	$|\mathcal{E}|$	$|\mathcal{V}^{(\mathcal{T})}|$	Average weight	Nodes density	Links density
LyonSchool	242	104	44,820	17,174	2.806	0.6824	0.0148
SFHH	403	127	17,223	10,815	4.079	0.2113	0.0017
LH10	76	321	7435	4880	4.448	0.2000	0.0081
Thiers13	327	246	35,862	32,546	5.256	0.4046	0.0027
InVS15	217	691	18,791	22,451	4.164	0.1497	0.0012
OpenABM-2k-100	2000	100	1,243,551	198,537	1.0	0.9927	0.0062
OpenABM-5k-20	5000	20	632,523	99,966	1.0	0.9997	0.0025

#### Baselines

We compare our approach with several baseline methods from the literature of time-varying graph embeddings, which learn time-stamped node representations: (1) DyANE [8], which learns temporal node embeddings with DeepWalk, mapping a time-varying graph into a supra-adjacency representation; (2) DynGEM [36], a deep autoencoder architecture which dynamically reconstructs each graph snapshot initializing model weights with parameters learned in previous time frames; (3) DynamicTriad [47], which captures structural information and temporal patterns of nodes, modeling the *triadic closure* process; (4) DySAT [45], a deep neural model that computes node embeddings by a joint self-attention mechanism applied on structural neighborhood and temporal dynamics; (5) ISGNS [39], an incremental skip-gram embedding model based on DeepWalk. Details about hyper-parameters used in each method can be found in Additional file 1.

### Downstream tasks

#### Node classification

The aim of this task is to classify nodes in epidemic states according to a SIR epidemic process with infection rate *β* and recovery rate *μ*. We simulated 30 realizations of the SIR process on top of each empirical graph with different combinations of parameters $(\beta ,\mu )$. We used similar combinations of epidemic parameters and the same dynamical process to produce SIR states as described in [8]. Then we set a logistic regression to classify epidemic states S-I-R assigned to each active node $i^{(k)}$ during the unfolding of the spreading process. We combine the embedding vectors of HOSGNS using the Hadamard (element-wise) product $\mathbf{w}_{i}\circ \mathbf{t}_{k}$. We compared with dynamic node embeddings learned from baselines. For fair comparison, all models produce time-stamped node representations with dimension $d = 128$ as input to the logistic regression.

#### Temporal event reconstruction

In this task, we aim to determine if a generic event $(i,j,k)$ (occurred or not) is in $\mathcal{H=(V,E,T)}$, i.e., if there is an edge between nodes *i* and *j* at time *k*. We create a random time-varying graph $\mathcal{H^{*}=(V,E^{*},T)}$ with same active nodes $\mathcal{V}^{(\mathcal{T})}$ and a number of $|\mathcal{E}|$ events that are not part of $\mathcal{E}$ (i.e. $\mathcal{E}\cap \mathcal{E}^{*} =$ Ø). In other words $\mathcal{E}^{*}$ contains random events that may occur only between the nodes that are active in each snapshot, disregarding other possible edges that involve inactive nodes. Embedding representations learned from $\mathcal{H}$ are used as features to train a logistic regression to predict if a given event $(i,j,k)$ is in $\mathcal{E}$ or in $\mathcal{E^{*}}$. We combine the embedding vectors of HOSGNS as follows: for $\mathrm{HOSGNS} ^{(\mathrm{stat})}$, we use the Hadamard product $\mathbf{w}_{i}\circ \mathbf{c}_{j}\circ \mathbf{t}_{k}$; for $\mathrm{HOSGNS} ^{(\mathrm{dyn})}$ and $\mathrm{HOSGNS} ^{(\mathrm{stat}|\mathrm{dyn})}$, we use $\mathbf{w}_{i}\circ \mathbf{c}_{j}\circ \mathbf{t}_{k}\circ \mathbf{s}_{k}$. For baseline methods, we aggregate vector embeddings to obtain link-level representations with binary operators (*Average*, *Hadamard*, *Weighted-L1*, *Weighted-L2* and *Concat*) as already used in previous works [19, 66]. For fair comparison, all models are required produce event representations with dimension $d = 192$.

#### Missing event prediction

In this task, we aim to predict the occurrence of an event $(i,j,k)$ *previously removed* from $\mathcal{H=(V,E,T)}$. We create a pruned time-varying graph $\mathcal{H^{\dagger}=(V,E^{\dagger},T)}$ with the same active nodes $\mathcal{V}^{(\mathcal{T})}$ and a number of events $|\mathcal{E}^{\dagger}| = 70\%~|\mathcal{E}|$ sampled from $\mathcal{H}$. Embedding representations learned from $\mathcal{H}^{\dagger}$ are used as features to train a logistic regression to predict missing occurred events $(i,j,k) \in \mathcal{E}\setminus \mathcal{E}^{\dagger}$ against the events $\mathcal{E}^{*}$ of a random time-varying graph $\mathcal{H^{*}=(V,E^{*},T)}$ (see above). We combine the embedding vectors of HOSGNS for the classification task as explained in the event reconstruction task.

### Results

In this section we first show downstream task performance results for the empirical and synthetic datasets, then we compare the different approaches in terms of training complexity, by measuring the number of trainable parameters and the training time with fixed number of training steps.

Tasks were evaluated using train-test split. To avoid information leakage from training to test, we randomly split $\mathcal{V}$ and $\mathcal{T}$ in train and test sets $(\mathcal{V}_{tr}, \mathcal{V}_{ts})$ and $(\mathcal{T}_{tr}, \mathcal{T}_{ts})$, with proportion 70%–30%. For node classification, only nodes in $\mathcal{V}_{tr}$ at times in $\mathcal{T}_{tr}$ were included in the train set, and only nodes in $\mathcal{V}_{ts}$ at times in $\mathcal{T}_{ts}$ were included in the test set. For event reconstruction and prediction, only events with $i,j \in \mathcal{V}_{tr}$ and $k \in \mathcal{T}_{tr}$ were included in the train set, and only events with $i,j \in \mathcal{V}_{ts}$ and $k \in \mathcal{T}_{ts}$ were included in the test set.

All approaches were evaluated for downstream tasks in terms of Macro-F1 scores in all datasets. 5 different runs of the embedding model are evaluated on 30 different train-test splits in every downstream tasks. We report the average score with standard error over all splits. In node classification, every SIR realization is assigned to a single embedding run to compute prediction scores. In event reconstruction and prediction tasks, a different random time-varying graph realization $\mathcal{H}^{*}$ to produce samples of non-occurring events is assigned to each train-test subset.

#### Empirical datasets

Results for the classification of nodes in epidemic states are shown in Table 2. We report here a subset of $(\beta ,\mu )$ but other combinations are available on Additional file 1. DynGEM and DynamicTriad have low scores, since they are not devised to learn from graph dynamics. Also DySAT has a bad performance in this task, since this method uses a context prediction objective that preserves the local structure without properly encoding dynamical patterns. $\mathrm{HOSGNS} ^{(\mathrm{stat})}$ is not able to capture the graph dynamics due to the static nature of $\boldsymbol{\mathcal{P}}^{(\mathrm{stat})}$. ISGNS, due to the incremental training, performs only marginally better than $\mathrm{HOSGNS} ^{(\mathrm{stat})}$. DyANE, $\mathrm{HOSGNS} ^{(\mathrm{stat}|\mathrm{dyn})}$ and $\mathrm{HOSGNS} ^{(\mathrm{dyn})}$ show good performance, with these two HOSGNS variants outperforming DyANE in most of the combinations of datasets and SIR parameters.

**Table 2:** Macro-F1 scores for classification of nodes in epidemic states according to different SIR epidemic processes over empirical datasets. For each $(\beta ,\mu )$ we highlight the two highest scores and underline the best one

(*β*,*μ*)	Model	Dataset				
		LyonSchool	SFHH	LH10	Thiers13	InVS15
(0.25,0.002)	DyANE	**78.1** ± **0.5**	67.0 ± 1.2	52.5 ± 1.7	**71.9** ± **0.6**	**64.3** ± **0.8**
	DynGEM	58.7 ± 2.8	35.9 ± 1.1	34.5 ± 0.7	35.5 ± 1.2	58.8 ± 1.1
	DynamicTriad	31.0 ± 0.4	28.8 ± 0.4	29.9 ± 0.3	30.3 ± 0.2	30.4 ± 0.2
	DySAT	27.3 ± 0.2	27.4 ± 0.3	29.7 ± 0.2	30.2 ± 0.2	30.5 ± 0.2
	ISGNS	63.5 ± 0.6	60.7 ± 0.8	54.1 ± 1.1	56.4 ± 0.6	52.3 ± 0.6
	$\text{HOSGNS} ^{(\text{stat})}$	55.5 ± 0.8	57.3 ± 1.1	45.9 ± 0.9	46.9 ± 0.7	44.5 ± 0.7
	$\text{HOSGNS} ^{(\text{dyn})}$	$\underline{\mathbf{79}\boldsymbol{.}\mathbf{2}}\pm \mathbf{0}\boldsymbol{.}\mathbf{5}$	$\underline{\mathbf{69}\boldsymbol{.}\mathbf{1}}\pm \mathbf{1}\boldsymbol{.}\mathbf{1}$	**59.6** ± **1.5**	71.8 ± 1.2	$\underline{\mathbf{64}\boldsymbol{.}\mathbf{6}}\pm \mathbf{0}\boldsymbol{.}\mathbf{7}$
	$\text{HOSGNS} ^{(\text{stat}|\text{dyn})}$	77.4 ± 0.6	**67.4** ± **1.2**	$\underline{\mathbf{59}\boldsymbol{.}\mathbf{7}}\pm \mathbf{1}\boldsymbol{.}\mathbf{2}$	$\underline{\mathbf{72}\boldsymbol{.}\mathbf{5}}\pm \mathbf{0}\boldsymbol{.}\mathbf{7}$	64.2 ± 1.0
(0.0625,0.002)	DyANE	72.2 ± 0.6	64.9 ± 1.7	**59.0** ± **1.2**	68.0 ± 0.5	$\underline{\mathbf{60}\boldsymbol{.}\mathbf{2}}\pm \mathbf{0}\boldsymbol{.}\mathbf{5}$
	DynGEM	56.4 ± 2.7	35.9 ± 4.1	35.8 ± 1.2	32.9 ± 1.2	55.0 ± 0.6
	DynamicTriad	29.5 ± 0.5	33.1 ± 2.5	29.6 ± 0.4	27.4 ± 0.3	28.4 ± 0.2
	DySAT	26.4 ± 0.2	29.5 ± 1.3	29.5 ± 0.3	26.5 ± 0.2	28.5 ± 0.2
	ISGNS	59.2 ± 0.3	57.1 ± 1.6	55.9 ± 1.0	49.0 ± 0.3	47.2 ± 0.3
	$\text{HOSGNS} ^{(\text{stat})}$	55.5 ± 0.7	57.6 ± 2.2	49.4 ± 0.8	45.5 ± 0.4	43.6 ± 0.5
	$\text{HOSGNS} ^{(\text{dyn})}$	$\underline{\mathbf{73}\boldsymbol{.}\mathbf{5}}\pm \mathbf{0}\boldsymbol{.}\mathbf{5}$	**65.7** ± **1.6**	$\underline{\mathbf{61}\boldsymbol{.}\mathbf{1}}\pm \mathbf{1}\boldsymbol{.}\mathbf{2}$	$\underline{\mathbf{69}\boldsymbol{.}\mathbf{5}}\pm \mathbf{0}\boldsymbol{.}\mathbf{3}$	**59.6** ± **0.5**
	$\text{HOSGNS} ^{(\text{stat}|\text{dyn})}$	**72.9** ± **0.6**	$\underline{\mathbf{66}\boldsymbol{.}\mathbf{3}}\pm \mathbf{1}\boldsymbol{.}\mathbf{9}$	58.2 ± 1.1	**68.5** ± **0.4**	59.0 ± 0.7
(0.1875,0.001)	DyANE	**74.7** ± **0.7**	67.7 ± 1.2	$\underline{\mathbf{63}\boldsymbol{.}\mathbf{4}}\pm \mathbf{1}\boldsymbol{.}\mathbf{8}$	72.7 ± 0.4	$\underline{\mathbf{68}\boldsymbol{.}\mathbf{6}}\pm \mathbf{0}\boldsymbol{.}\mathbf{4}$
	DynGEM	57.4 ± 2.8	36.2 ± 2.6	41.4 ± 1.3	34.8 ± 1.3	61.2 ± 0.9
	DynamicTriad	32.3 ± 0.5	31.5 ± 0.8	30.5 ± 0.4	27.9 ± 0.3	30.0 ± 0.2
	DySAT	26.4 ± 0.2	29.4 ± 0.8	30.0 ± 0.3	27.7 ± 0.3	29.9 ± 0.2
	ISGNS	65.1 ± 0.5	63.0 ± 1.4	60.2 ± 1.7	56.0 ± 0.5	52.5 ± 0.5
	$\text{HOSGNS} ^{(\text{stat})}$	56.9 ± 0.8	59.4 ± 1.7	48.5 ± 1.1	49.0 ± 0.6	46.2 ± 0.8
	$\text{HOSGNS} ^{(\text{dyn})}$	$\underline{\mathbf{76}\boldsymbol{.}\mathbf{5}}\pm \mathbf{0}\boldsymbol{.}\mathbf{4}$	**68.6** ± **1.1**	62.4 ± 1.7	$\underline{\mathbf{74}\boldsymbol{.}\mathbf{8}}\pm \mathbf{0}\boldsymbol{.}\mathbf{5}$	**67.9** ± **0.7**
	$\text{HOSGNS} ^{(\text{stat}|\text{dyn})}$	74.5 ± 0.4	$\underline{\mathbf{69}\boldsymbol{.}\mathbf{4}}\pm \mathbf{1}\boldsymbol{.}\mathbf{4}$	**62.5** ± **2.0**	**73.6** ± **0.6**	67.3 ± 0.5

Results for the temporal event reconstruction task are reported in Table 3. Temporal event reconstruction is not performed well by DynGEM. DynamicTriad has better performance with Weighted-L1 and Weighted-L2 operators, while DyANE, DySAT and ISGNS have better performance using Hadamard and Weighted-L2. ISGNS has the second best perfomances in most of the datasets. Since Hadamard product is explicitly used in Eq. (8) to optimize HOSGNS, all HOSGNS variants show best scores with this operator. $\mathrm{HOSGNS} ^{(\mathrm{stat})}$ outperforms all approaches, setting new state-of-the-art results in this task. The $\boldsymbol{\mathcal{P}}^{(\mathrm{dyn})}$ representation used as input to $\mathrm{HOSGNS} ^{(\mathrm{dyn})}$ does not focus on events but on dynamics, so the performance for event reconstruction is slightly below DyANE, while $\mathrm{HOSGNS} ^{(\mathrm{stat}|\mathrm{dyn})}$ is comparable to DyANE.

**Table 3:** Macro-F1 scores for temporal event reconstruction in empirical datasets. We highlight in bold the two best scores for each dataset. For baseline models we underline their highest score

Model	Operator	Dataset				
		LyonSchool	SFHH	LH10	Thiers13	InVS15
DyANE	Average	56.4 ± 0.4	52.9 ± 0.5	52.3 ± 0.6	51.0 ± 0.4	52.7 ± 0.4
	Hadamard	89.7 ± 0.3	$\underline{86.5}\pm 0.3$	$\underline{74.6}\pm 0.6$	94.7 ± 0.1	94.1 ± 0.1
	Weighted-L1	90.2 ± 0.2	83.3 ± 0.5	73.3 ± 0.7	94.7 ± 0.1	94.4 ± 0.2
	Weighted-L2	$\underline{90.6}\pm 0.2$	84.5 ± 0.5	72.0 ± 0.5	$\underline{95.0}\pm 0.1$	$\underline{94.8}\pm 0.2$
	Concat	65.7 ± 0.4	53.8 ± 0.4	56.2 ± 0.6	57.0 ± 0.4	50.9 ± 0.4
DynGEM	Average	57.7 ± 0.5	56.8 ± 0.7	$\underline{54.8}\pm 1.5$	40.4 ± 1.5	42.8 ± 0.9
	Hadamard	$\underline{62.2}\pm 0.4$	55.1 ± 1.0	52.5 ± 1.6	40.8 ± 1.5	43.7 ± 1.0
	Weighted-L1	58.4 ± 0.6	52.3 ± 0.7	50.9 ± 1.2	$\underline{41.3}\pm 1.6$	44.8 ± 0.9
	Weighted-L2	53.7 ± 0.6	47.0 ± 0.8	47.0 ± 1.3	39.2 ± 1.2	43.6 ± 0.6
	Concat	60.4 ± 0.4	$\underline{57.8}\pm 0.3$	48.9 ± 1.7	36.9 ± 1.3	$\underline{45.7}\pm 1.0$
DynamicTriad	Average	51.7 ± 0.2	56.9 ± 0.4	60.2 ± 0.6	58.1 ± 0.2	56.1 ± 0.3
	Hadamard	60.3 ± 0.3	58.9 ± 0.4	59.5 ± 0.5	62.2 ± 0.3	64.7 ± 0.3
	Weighted-L1	$\underline{79.1}\pm 0.4$	72.3 ± 0.4	75.5 ± 0.6	70.8 ± 0.3	78.1 ± 0.2
	Weighted-L2	77.4 ± 0.4	$\underline{73.4}\pm 0.4$	$\underline{77.4}\pm 0.5$	$\underline{72.4}\pm 0.2$	$\underline{78.9}\pm 0.3$
	Concat	52.2 ± 0.2	53.4 ± 0.3	55.9 ± 0.7	55.1 ± 0.2	53.2 ± 0.3
DySAT	Average	51.1 ± 0.3	49.6 ± 0.4	51.6 ± 0.5	50.4 ± 0.2	50.1 ± 0.3
	Hadamard	$\underline{75.1}\pm 0.5$	$\underline{52.9}\pm 0.3$	54.8 ± 0.6	$\underline{71.1}\pm 0.4$	$\underline{66.8}\pm 0.5$
	Weighted-L1	72.4 ± 0.5	51.5 ± 0.3	56.1 ± 0.6	66.4 ± 0.4	64.8 ± 0.3
	Weighted-L2	72.4 ± 0.5	51.7 ± 0.3	$\underline{56.8}\pm 0.7$	66.5 ± 0.4	63.7 ± 0.4
	Concat	50.0 ± 0.3	50.1 ± 0.4	52.3 ± 0.5	49.8 ± 0.2	50.9 ± 0.3
ISGNS	Average	53.4 ± 0.4	50.3 ± 0.5	48.1 ± 0.6	49.4 ± 0.4	45.9 ± 0.5
	Hadamard	$\underline{90.1}\pm 0.3$	87.2 ± 0.4	80.8 ± 0.7	96.7 ± 0.2	96.7 ± 0.2
	Weighted-L1	89.9 ± 0.3	87.7 ± 0.4	81.6 ± 0.4	96.8 ± 0.2	96.4 ± 0.2
	Weighted-L2	89.7 ± 0.3	$\underline{\mathbf{88}\boldsymbol{.}\mathbf{2}}\pm \mathbf{0}\boldsymbol{.}\mathbf{4}$	$\underline{\mathbf{81}\boldsymbol{.}\mathbf{7}}\pm \mathbf{0}\boldsymbol{.}\mathbf{5}$	$\underline{\mathbf{96}\boldsymbol{.}\mathbf{9}}\pm \mathbf{0}\boldsymbol{.}\mathbf{1}$	$\underline{\mathbf{96}\boldsymbol{.}\mathbf{8}}\pm \mathbf{0}\boldsymbol{.}\mathbf{2}$
	Concat	57.1 ± 0.5	50.2 ± 0.4	48.8 ± 0.7	52.7 ± 0.4	43.8 ± 0.4
$\text{HOSGNS} ^{(\text{stat})}$	Hadamard	$\mathbf{{98}\boldsymbol{.}\mathbf{5}}\pm \mathbf{0}\boldsymbol{.}\mathbf{1}$	$\mathbf{{98}\boldsymbol{.}\mathbf{8}}\pm \mathbf{0}\boldsymbol{.}\mathbf{1}$	$\mathbf{{99}\boldsymbol{.}\mathbf{8}}\pm \mathbf{0}\boldsymbol{.}\mathbf{1}$	$\mathbf{{99}\boldsymbol{.}\mathbf{6}}\pm \mathbf{0}\boldsymbol{.}\mathbf{1}$	$\mathbf{{99}\boldsymbol{.}\mathbf{1}}\pm \mathbf{0}\boldsymbol{.}\mathbf{1}$
$\text{HOSGNS} ^{(\text{dyn})}$	Hadamard	90.3 ± 0.2	80.9 ± 0.4	68.1 ± 0.7	93.5 ± 0.2	87.2 ± 0.2
$\text{HOSGNS} ^{(\text{stat}|\text{dyn})}$	Hadamard	**91.8** ± **0.2**	86.7 ± 0.4	73.6 ± 0.6	94.3 ± 0.1	89.0 ± 0.2

Table 4 outlines the results for the missing event prediction task. In this case $\mathrm{HOSGNS} ^{(\mathrm{stat})}$ has lower performance, but comparable with DynGEM and DynamicTriad. DySAT and ISGNS work slightly better with Hadamard or Weighted-L1/L2 operator, but they are outperformed by DyANE that has an excellent performance with Hadamard or Weighted-L2. However $\mathrm{HOSGNS} ^{(\mathrm{dyn})}$ and $\mathrm{HOSGNS} ^{(\mathrm{stat}|\mathrm{dyn})}$ have the best scores, which emphasize the importance of leveraging dynamics to learn and predict missing information.

**Table 4:** Macro-F1 scores for missing event prediction in empirical datasets. We highlight in bold the two best scores for each dataset. For baseline models we underline their highest score

Model	Operator	Dataset				
		LyonSchool	SFHH	LH10	Thiers13	InVS15
DyANE	Average	56.8 ± 0.6	50.6 ± 0.8	51.3 ± 1.0	49.1 ± 0.6	49.3 ± 0.8
	Hadamard	87.3 ± 0.3	73.5 ± 0.6	$\underline{67.0}\pm 1.0$	$\underline{87.2}\pm 0.3$	$\underline{80.1}\pm 0.8$
	Weighted-L1	87.8 ± 0.3	73.3 ± 0.6	65.9 ± 1.0	84.0 ± 0.4	78.4 ± 0.6
	Weighted-L2	$\underline{88.5}\pm 0.2$	$\underline{73.7}\pm 0.5$	66.1 ± 1.0	84.4 ± 0.4	78.9 ± 0.6
	Concat	64.4 ± 0.5	52.4 ± 0.8	51.9 ± 1.0	57.0 ± 0.6	51.4 ± 0.7
DynGEM	Average	56.2 ± 0.5	$\underline{51.8}\pm 0.8$	$\underline{52.0}\pm 1.1$	49.7 ± 0.5	50.9 ± 0.7
	Hadamard	54.8 ± 0.6	51.3 ± 0.7	51.7 ± 1.2	44.7 ± 0.7	$\underline{50.9}\pm 0.6$
	Weighted-L1	55.5 ± 0.4	48.5 ± 0.8	50.2 ± 1.0	$\underline{52.2}\pm 0.4$	49.8 ± 0.7
	Weighted-L2	53.2 ± 0.7	47.8 ± 0.9	48.0 ± 1.1	48.9 ± 0.6	45.3 ± 0.6
	Concat	$\underline{58.2}\pm 0.5$	50.4 ± 0.8	46.4 ± 1.4	48.8 ± 0.5	49.9 ± 0.6
DynamicTriad	Average	51.4 ± 0.4	52.6 ± 0.6	53.0 ± 0.8	52.0 ± 0.4	49.9 ± 0.7
	Hadamard	53.1 ± 0.4	49.5 ± 0.6	52.0 ± 0.8	51.7 ± 0.5	49.8 ± 0.6
	Weighted-L1	64.3 ± 0.4	56.6 ± 0.7	54.2 ± 0.9	53.6 ± 0.4	47.2 ± 0.6
	Weighted-L2	$\underline{64.5}\pm 0.4$	$\underline{57.3}\pm 0.7$	$\underline{54.9}\pm 0.9$	$\underline{54.5}\pm 0.5$	47.0 ± 0.6
	Concat	52.6 ± 0.3	51.8 ± 0.5	52.7 ± 0.9	51.5 ± 0.3	$\underline{49.9}\pm 0.6$
DySAT	Average	51.3 ± 0.4	51.6 ± 0.6	52.5 ± 0.8	50.0 ± 0.4	50.3 ± 0.6
	Hadamard	$\underline{73.8}\pm 0.6$	$\underline{52.5}\pm 0.7$	56.6 ± 0.7	$\underline{68.5}\pm 0.5$	61.5 ± 0.8
	Weighted-L1	71.3 ± 0.5	52.0 ± 0.6	$\underline{57.6}\pm 0.8$	63.2 ± 0.6	$\underline{64.4}\pm 0.5$
	Weighted-L2	70.7 ± 0.5	51.5 ± 0.7	56.5 ± 0.8	63.1 ± 0.5	63.4 ± 0.5
	Concat	49.2 ± 0.4	48.8 ± 0.8	52.4 ± 0.9	49.8 ± 0.5	50.4 ± 0.6
ISGNS	Average	52.4 ± 0.6	49.5 ± 0.8	44.9 ± 0.9	48.0 ± 0.4	42.7 ± 0.8
	Hadamard	79.8 ± 0.4	59.3 ± 0.7	61.1 ± 1.2	59.3 ± 0.6	$\underline{51.7}\pm 0.7$
	Weighted-L1	80.8 ± 0.3	59.8 ± 0.7	61.7 ± 1.0	59.0 ± 0.6	49.8 ± 0.7
	Weighted-L2	$\underline{81.5}\pm 0.3$	$\underline{60.2}\pm 0.7$	$\underline{62.5}\pm 0.9$	$\underline{59.9}\pm 0.6$	51.5 ± 0.7
	Concat	55.8 ± 0.7	50.8 ± 0.6	46.8 ± 0.8	52.2 ± 0.5	48.5 ± 0.6
$\text{HOSGNS} ^{(\text{stat})}$	Hadamard	52.1 ± 0.4	43.8 ± 0.6	34.2 ± 0.2	55.9 ± 0.6	43.0 ± 0.5
$\text{HOSGNS} ^{(\text{dyn})}$	Hadamard	**89.2** ± **0.2**	**74.9** ± **0.6**	**67.1** ± **0.8**	**90.7** ± **0.3**	**81.4** ± **0.5**
$\text{HOSGNS} ^{(\text{stat}|\text{dyn})}$	Hadamard	**89.2** ± **0.3**	**76.3** ± **0.7**	**68.5** ± **1.0**	**89.9** ± **0.3**	**80.8** ± **0.6**

Results for HOSGNS models using other operators are available in Additional file 1. We observe an overall good performance of $\mathrm{HOSGNS} ^{(\mathrm{stat}|\mathrm{dyn})}$ in all downstream tasks, being in almost all cases among the two highest scores, compared to the other two HOSGNS variants which excel in certain tasks but have lower performance in the others.

#### Synthetic datasets

Here we report the performance of downstream tasks with the two synthetic datasets only for $\mathrm{HOSGNS} ^{(\mathrm{stat})}$ and $\mathrm{HOSGNS} ^{(\mathrm{dyn})}$, given the similar performance of $\mathrm{HOSGNS} ^{(\mathrm{dyn})}$ and $\mathrm{HOSGNS} ^{(\mathrm{stat}|\mathrm{dyn})}$ in previous experiments. We also chose DyANE as the only baseline, given its better performance compared to other baselines in empirical datasets.

Results for the node classification task for a set of $(\beta ,\mu )$ combinations are reported in Table 5, with other combinations available in Additional file 1. These results reflect previous results on empirical datasets, with $\mathrm{HOSGNS} ^{(\mathrm{dyn})}$ performance comparable or superior to DyANE.

**Table 5:** Macro-F1 scores for classification of nodes in epidemic states according to different SIR epidemic processes for synthetic datasets. For each $(\beta ,\mu )$ we highlight the best score

(*β*,*μ*)	Model	Dataset	
		OpenABM-2k-100	OpenABM-5k-20
(0.25,0.002)	DyANE	**57.9** ± **1.8**	59.6 ± 1.7
	$\text{HOSGNS} ^{(\text{stat})}$	31.2 ± 0.1	27.8 ± 0.6
	$\text{HOSGNS} ^{(\text{dyn})}$	57.5 ± 1.8	**61.0** ± **1.1**
(0.0625,0.002)	DyANE	**61.8** ± **0.4**	53.8 ± 1.3
	$\text{HOSGNS} ^{(\text{stat})}$	29.8 ± 0.2	29.4 ± 1.4
	$\text{HOSGNS} ^{(\text{dyn})}$	59.5 ± 0.9	**54.5** ± **1.4**
(0.1875,0.001)	DyANE	60.3 ± 1.4	59.6 ± 1.5
	$\text{HOSGNS} ^{(\text{stat})}$	31.9 ± 0.2	27.4 ± 0.7
	$\text{HOSGNS} ^{(\text{dyn})}$	**60.5** ± **1.1**	**60.9** ± **1.0**

Results for the event reconstruction and prediction tasks are reported in Table 6. DyANE performs well with Hadamard operation, but nevertheless the scores are below $\mathrm{HOSGNS} ^{(\mathrm{dyn})}$ and $\mathrm{HOSGNS} ^{(\mathrm{stat})}$ scores. Especially with $\mathrm{HOSGNS} ^{(\mathrm{stat})}$, the performance of event reconstruction is not much larger than even prediction, contrary to empirical datasets. This difference might be due to the different topological features of synthetic networks respect to empirical ones.

**Table 6:** Macro-F1 scores in temporal event reconstruction and missing event prediction for synthetic datasets. We highlight in bold the best two scores for each dataset. For baseline model we underline their highest score

Model	Operator	Dataset			
		OpenABM-2k-100		OpenABM-5k-20	
		Reconstruction	Prediction	Reconstruction	Prediction
DyANE	Average	52.2 ± 0.1	51.7 ± 0.1	51.9 ± 0.1	51.9 ± 0.1
	Hadamard	$\underline{76.4}\pm 0.1$	$\underline{72.4}\pm 0.2$	$\underline{\mathbf{90}}\boldsymbol{.}\mathbf{5}\pm \mathbf{0}\boldsymbol{.}\mathbf{3}$	$\underline{77.8}\pm 0.2$
	Weighted-L1	70.3 ± 0.1	67.4 ± 0.2	78.2 ± 0.7	70.5 ± 0.3
	Weighted-L2	70.3 ± 0.1	67.7 ± 0.1	78.8 ± 0.5	70.9 ± 0.3
	Concat	53.8 ± 0.1	54.6 ± 0.1	52.5 ± 0.1	52.5 ± 0.2
$\text{HOSGNS} ^{(\text{stat})}$	Hadamard	**91.1** ± **0.1**	**87.0** ± **0.1**	**98.7** ± **0.1**	**86.0** ± **0.1**
$\text{HOSGNS} ^{(\text{dyn})}$	Hadamard	**78.7** ± **0.1**	**79.8** ± **0.2**	82.8 ± 0.3	**82.4** ± **0.2**

#### Training complexity

We report in Table 7 the number of trainable parameters and training time duration for each considered algorithm, when applied to an empirical graph (LyonSchool) and to the synthetic ones. The proposed HOSGNS model requires a number of trainable parameters that is orders of magnitude smaller than other approaches, with a training time considerably shorter as the number of nodes increases, given a fixed number of training iterations. ISGNS has a comparable number of parameters because it incrementally updates $\mathcal{O}(|\mathcal{V}|)$ parameters moving across the $|\mathcal{T}|$ snapshots. DySAT training time is considerably higher due to the computational overhead of the self-attention mechanism.

**Table 7:** Number of trainable parameters and training time of each time-varying graph representation learning model for LyonSchool and the two synthetic datasets. The embedding dimension is fixed to 128, technical specifications of the computing system and hyper-parameters configuration are reported in Additional file 1

Model	Dataset					
	LyonSchool		OpenABM-2k-100		OpenABM-5k-20	
	$|\mathcal{V}|=242$, $|\mathcal{T}|=104$		$|\mathcal{V}|=2000$, $|\mathcal{T}|=100$		$|\mathcal{V}|=5000$, $|\mathcal{T}|=20$	
	Tr. parameters	Tr. time	Tr. parameters	Tr. time	Tr. parameters	Tr. time
DyANE	4,396,544	$ 62~\text{s} $	50,825,472	$ 1014~\text{s} $	25,591,296	$ 448~\text{s} $
DynGEM	459,270	$ 516~\text{s} $	1,867,428	$ 10\text{,}765~\text{s} $	4,270,428	$ 23\text{,}307~\text{s} $
DynamicTriad	3,221,632	$ 1131~\text{s} $	25,600,128	$ 17\text{,}191~\text{s} $	12,800,128	$ 12\text{,}625~\text{s} $
DySAT	98,336	$ 18\text{,}323~\text{s} $	323,232	$ 152\text{,}976~\text{s} $	707,232	$ 8958~\text{s} $
ISGNS	61,952	$ 381~\text{s} $	512,000	$ 5895~\text{s} $	1,280,000	$ 3062~\text{s} $
$\text{HOSGNS} ^{(\text{stat})}$	75,264	$ 316~\text{s} $	524,800	$ 548~\text{s} $	1,282,560	$ 724~\text{s} $
$\text{HOSGNS} ^{(\text{dyn})}$	88,576	$ 303~\text{s} $	537,600	$ 565~\text{s} $	1,285,120	$ 734~\text{s} $

### Embedding space visualization

One of the main advantages of HOSGNS is that it is able to disentangle the role of nodes and time by learning representations of nodes and time intervals separately. In this section, we include plots with two-dimensional projections of these embeddings, made with UMAP [67] for manifold learning and non-linear dimensionality reduction. With these plots, we show that the embedding matrices learned by $\mathrm{HOSGNS} ^{(\mathrm{stat})}$ and $\mathrm{HOSGNS} ^{(\mathrm{dyn})}$ successfully capture both the structure and the dynamics of the time-varying graph.

Dynamical information can be represented by associating each embedding vector to its corresponding time interval $k \in \mathcal{T}$, and graph structure can be represented by associating each embedding vector to a community membership. While community membership can be estimated by different community detection methods, we choose to use a dataset with ground truth data containing node membership information. We consider the LyonSchool dataset as a case study, widely investigated in literature respect to structural and spreading properties [68–73]. This dataset spans two days and includes metadata (Table 8) concerning the class of each participant of the school (10 different labels for children and 1 label for teachers), and we identify the community membership of each individual according to these labels (*class* labels). Moreover we also assign *time* labels according to activation of individual nodes in temporal snapshots.

**Table 8:** Number of class components for each labelled class in LyonSchool dataset

Class name	Class label	Number of children or teachers
CP-A	0	23
CP-B	1	25
CE1-A	2	23
CE1-B	3	26
CE2-A	4	23
CE2-B	5	22
CM1-A	6	21
CM1-B	7	23
CM2-A	8	22
CM2-B	9	24
Teachers	10	10

**Figure 3:**
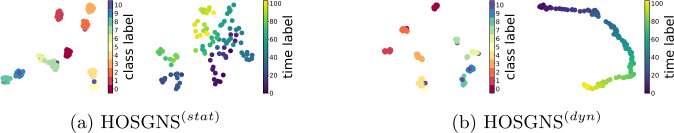
Two-dimensional projections of the 128-dim embedding manifold spanned by embedding matrices **W** (left of each panel) and **T** (right of each panel), trained on LyonSchool data, of HOSGNS model trained on: (**a**) $\boldsymbol{\mathcal{P}}^{(\text{stat})}$ and (**b**) $\boldsymbol{\mathcal{P}}^{(\text{dyn})}$. These plots show how the community structure and the evolution of time is captured by individual node embeddings $\{\mathbf{w}_{i}\}_{i \in \mathcal{V}}$ and time embeddings $\{\mathbf{t}_{k}\}_{k \in \mathcal{T}}$

To show how disentangled representations capture different aspects of the evolving graph, in Fig. 3 we plot individual representations of nodes $i \in \mathcal{V}$ and time slices $k \in \mathcal{T}$ labeled according to the class membership and the time snapshot respectively. Both $\mathrm{HOSGNS} ^{(\mathrm{stat})}$ and $\mathrm{HOSGNS} ^{(\mathrm{dyn})}$ capture the community structure (left of each panel) with node embeddings clustered into the ground-truth classes, but dynamical information expressed by time embeddings (right of each panel) is different for the two methods. Due to the time-respecting topology of the supra-adjacency graph, $\mathrm{HOSGNS} ^{(\mathrm{dyn})}$ captures the causality of node co-occurrences encoding temporal slices into a time-ordered one-dimensional manifold. $\mathrm{HOSGNS} ^{(\mathrm{stat})}$ is built on the snapshot representation, invariant over time permutation, and thus the temporal encoding is constrained to the local connectivity structure of graph slices.

**Figure 4:**
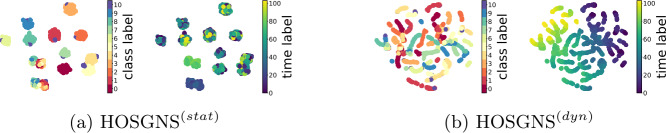
Two-dimensional projections of the 128-dim embedding manifold spanned by dynamic node embeddings, trained on LyonSchool data and obtained with Hadamard products $\{\mathbf{w}_{i}\circ \mathbf{t}_{k}\}_{(i,k) \in \mathcal{V}^{( \mathcal{T})}}$ between rows of **W** (node embeddings) and **T** (time embeddings), from HOSGNS model trained on: (**a**) $\boldsymbol{\mathcal{P}}^{(\text{stat})}$ and (**b**) $\boldsymbol{\mathcal{P}}^{(\text{dyn})}$. We highlight the temporal participation to communities (left of each panel) and the time interval of activation (right of each panel)

In Fig. 4 we visualize representations of temporal nodes $i^{(k)} \in \mathcal{V}^{(\mathcal{T})}$, computed as Hadamard products of nodes and time embeddings. $\mathrm{HOSGNS} ^{(\mathrm{stat})}$ projections show clusters of nodes active at multiple times representing different social situations: interactions during lectures present uniform class labels and heterogeneous time labels, whereas interactions occurred in social spaces with mixed classes present uniform time labels and heterogeneous class labels. This is in line with previous studies [13], where different patterns of interactions are found during school activities, and gatherings in social spaces (such as canteen and playground) are more concentrated during lunch time. $\mathrm{HOSGNS} ^{(\mathrm{dyn})}$ projected embeddings, due to the causality information encoded in time representations, display trajectories of social interactions that span over time in the embedding space, with communities interacting and mixing at different points of the day.

**Figure 5:**
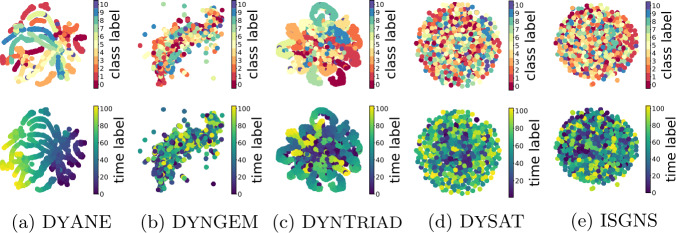
Two-dimensional projections of the 128-dim embedding manifold spanned by dynamic node embeddings for LyonSchool data learned with baseline methods. As in Fig. 4 we highlight the temporal participation to communities (top of each panel) and the time interval of activation (bottom of each panel)

In Fig. 5 we see dynamic node embeddings computed with baseline methods without dissociating structure and time. The embedding space in DyANE encodes properly the time-aware topology, since the model is based on the supra-adjacency graph like $\mathrm{HOSGNS} ^{(\mathrm{dyn})}$. Also DynamicTriad captures significant temporal structures, but it is less effective to express the overall dynamics since it is limited in modeling the triadic closure process. Other relevant interaction patterns are instead accounted with supra-adjacency random walks. DynGEM, DySAT and ISGNS embedding spaces do not encode any structural or temporal information.

## Conclusions

In this paper, we introduce Higher-Order Skip-Gram with Negative Sampling (HOSGNS) for time-varying graph representation learning. We generalize the skip-gram embedding approach that implicitly performs a factorization of the shifted PMI matrix to perform implicit factorization of a shifted PMI tensor. We show how to optimize HOSGNS for the generic *n*th-order case, and how to apply 3rd-order and 4th-order SGNS on different higher-order representations of time-varying graphs.

The embedding representations learned by HOSGNS outperform other methods in the literature and set new state-of-the-art results for solving downstream tasks. By learning embeddings on empirical time-resolved face-to-face proximity data, such representations can be effectively used to predict the outcomes of a SIR spreading process over the time-varying graph. They also can be effectively used for network reconstruction and link prediction.

HOSGNS is able to learn more compact representations of time-varying graphs due to the reduced number of parameters, with computational complexity that is comparable or lower than other state-of-the-art methods. By learning disentangled representations of nodes and time intervals, HOSGNS uses a number of parameters in the order of $\mathcal{O}(|\mathcal{V}| + |\mathcal{T}|)$, while models that learn node-time representations need a number of parameters that is at least $\mathcal{O}(|\mathcal{V}|\cdot |\mathcal{T}|)$.

While other methods such as DyANE assume that the whole temporal network has to be known, here we relax this assumption and we show that the learned representations can be used also for predicting events that are not seen during the representation learning phase. Yet, one limitation still holds: the transductivity of the model makes it unable to generalize the embedding representations outside the set of observed temporal slices. A future work to tackle this limitation is the extension of the methodology to include prior constraints, such as temporal smoothness and stability of embeddings over consecutive time slices, or to equip the model with an inductive framework.

We show that HOSGNS can be intuitively applied to time-varying graphs, but this methodology can be easily adapted to solve other representation learning problems that involve multi-modal data and multi-layered graph representations, where the purpose is to factorize higher-order dependencies between elementary units of the system. Beyond these applications, extensions of the model can find usage in feature learning on higher-order systems, i.e. hypergraphs and simplicial complexes, where interactions among vertices are intrinsically polyadic.

## Supplementary Information

Below is the link to the electronic supplementary material.

Supplementary Material. Supplementary Material include formal proofs and additional experiments not shown in the manuscript. (PDF 2.0 MB)

## Data Availability

We use open data which can be downloaded on http://www.sociopatterns.org or generated from https://github.com/BDI-pathogens/OpenABM-Covid19. The source code is publicly available at https://github.com/simonepiaggesi/hosgns.
