# Supplementary material for: Time-varying graph representation learning via higher-order skip-gram with negative sampling
Source: EPJ Data Sci. 2022 May 28;11(1):33. doi: 10.1140/epjds/s13688-022-00344-8 (PMC9143726; doi:10.1140/epjds/s13688-022-00344-8)
Supplement: Supplementary file 1 — Supplementary Material. Supplementary Material include formal proofs and additional experiments not shown in the manuscript. (PDF 2.0 MB) [file 13688_2022_344_MOESM1_ESM.pdf]

Time-varying Graph Representation Learning via Higher Order Skip-Gram with Negative Sampling

Simone Piaggesi
ISI Foundation, Turin, Italy
University of Bologna, Bologna, Italy
simone.piaggesi2@unibo.it

André Panisson
CENTAI, Turin, Italy
andre.panisson@centai.eu

A Supplementary Information

A.1 Low-rank tensor decomposition

Low-rank tensor decomposition [1] aims to factorize a generic tensor into a sum of rank-one tensors. For example, given a 3rd-order tensor $\boldsymbol{\mathcal{X}} \in \mathbb{R}^{I\times J\times K}$, the rank-$R$ decomposition of $\boldsymbol{\mathcal{X}}$ takes the form of a ternary product between three factor matrices:

$$\boldsymbol{\mathcal{X}} \approx [\![\mathbf{A},\mathbf{B},\mathbf{C}]\!] \equiv \sum_{r=1}^{R} \mathbf{a}_{:r}\otimes \mathbf{b}_{:r}\otimes \mathbf{c}_{:r} \tag{A.1}$$

where $\mathbf{a}_{:r}\in\mathbb{R}^{I}$, $\mathbf{b}_{:r}\in\mathbb{R}^{J}$ and $\mathbf{c}_{:r}\in\mathbb{R}^{K}$ are the columns of the latent factor matrices $\mathbf{A}\in\mathbb{R}^{I\times R}$, $\mathbf{B}\in\mathbb{R}^{J\times R}$ and $\mathbf{C}\in\mathbb{R}^{K\times R}$ and $\otimes$ denotes the outer product. When R is the rank of $\boldsymbol{\mathcal{X}}$, Eq. (A.1) holds with an equality, and the above operation is called Canonical Polyadic (CP) decomposition. Elementwise the previous relation is written as:

$$(\boldsymbol{\mathcal{X}})_{ijk} \approx [\![\mathbf{a}_i,\mathbf{b}_j,\mathbf{c}_k]\!] \equiv \sum_{r=1}^{R} \mathbf{A}_{ir}\mathbf{B}_{jr}\mathbf{C}_{kr} \tag{A.2}$$

where $\mathbf{a}_i$, $\mathbf{b}_j$, $\mathbf{c}_k\in\mathbb{R}^R$ are rows of the factor matrices. For 2nd-order tensors (matrices) the operation is equivalent to the low-rank matrix decomposition ($\mathbf{X}\approx\mathbf{A}\mathbf{B}^{\mathrm{T}}$).
For a generic Nth-order tensor $\boldsymbol{\mathcal{X}}\in\mathbb{R}^{I_1\times I_2\times\cdots\times I_N}$, low-rank decomposition is expressed as:

$$(\boldsymbol{\mathcal{X}})_{i_1 i_2\ldots i_N} \approx [\![\mathbf{a}^{(1)}_{i_1},\mathbf{a}^{(2)}_{i_2},\ldots,\mathbf{a}^{(N)}_{i_N}]\!] \equiv \sum_{r=1}^{R} \mathbf{A}^{(1)}_{i_1 r}\,\mathbf{A}^{(2)}_{i_2 r}\ldots\mathbf{A}^{(N)}_{i_N r} \tag{A.3}$$

where $\mathbf{a}^{(1)}_{i_1},\mathbf{a}^{(2)}_{i_2},\ldots,\mathbf{a}^{(N)}_{i_N}\in\mathbb{R}^R$ ($i_n\in\{1,\ldots,I_n\},\ n\in\{1,\ldots,N\}$) are rows of factor matrices $\mathbf{A}^{(1)}\in\mathbb{R}^{I_1\times R}$, $\mathbf{A}^{(2)}\in\mathbb{R}^{I_2\times R}$, $\ldots$, $\mathbf{A}^{(N)}\in\mathbb{R}^{I_N\times R}$.

A.2 Higher-order skip-gram with negative sampling as implicit tensor factorization

A.2.1 Derivation of the objective function

Here we address the problem of generalizing SGNS to learn embedding representations from higher-order co-occurrences.
We consider a set of training samples $\mathcal{D}=\{(i_1,i_2,\ldots,i_N):\ i_1\in\mathcal{V}_1,\ i_2\in\mathcal{V}_2,\ \ldots,\ i_N\in\mathcal{V}_N\}$ obtained by collecting co-occurrences among elements from N sets $\mathcal{V}_1,\mathcal{V}_2,\ldots,\mathcal{V}_N$. While SGNS is limited to pairs of node-context (i,j), here $\mathcal{D}$ is constructed with three (or more) variables, e.g. sampling random walks over a higher-order data structure. We denote as $\#(i_1,i_2,\ldots,i_N)$ the number of times the tuple $(i_1,i_2,\ldots,i_N)$

appears in $\mathcal{D}$. Similarly we use $\#i_n = \sum_{i_1..i_{n-1}i_{n+1}..i_N} \#(i_1,..,i_n,..,i_N)$ as the number of times each distinct element occurs in $\mathcal{D}$, with relative frequencies $P_{\mathcal{D}}(i_1,\dots,i_N) = \frac{\#(i_1,\dots,i_N)}{|\mathcal{D}|}$ and $P_{\mathcal{D}}(i_n) = \frac{\#(i_n)}{|\mathcal{D}|}$.
Optimization is performed as a binary classification task, where the objective is to discern occurrences actually coming from $\mathcal{D}$ from random occurrences. We define the likelihood for a single observation $(i_1,\dots,i_N) \in \mathcal{D}$ by applying a sigmoid to the higher-order inner product $[\![\cdot]\!]$ of corresponding d-dimensional representations:

$$P[\ (i_1,\dots,i_N) \in \mathcal{D} \mid \mathbf{a}^{(1)}_{i_1},\dots,\mathbf{a}^{(N)}_{i_N}\ ] = \sigma\big(\ [\![\mathbf{a}^{(1)}_{i_1},\dots,\mathbf{a}^{(N)}_{i_N}]\!]\ \big) \equiv \sigma\left(\ \sum_{r=1}^{d} \mathbf{A}^{(1)}_{i_1 r}\ \mathbf{A}^{(2)}_{i_2 r}\dots\mathbf{A}^{(N)}_{i_N r}\right) \quad \text{(A.4)}$$

where we have N trainable embedding matrices $\mathbf{A}^{(1)} \in \mathbb{R}^{|\mathcal{V}_1|\times d},\dots,\mathbf{A}^{(N)} \in \mathbb{R}^{|\mathcal{V}_N|\times d}$ and each embedding vector $\mathbf{a}^{(n)}_{i_n}$ is the i_n-th row of the matrix $\mathbf{A}^{(n)}$.
We define the loss with negative sampling fixing i_1 and picking negative tuples $(\nu_2,\dots,\nu_N)$ according to the noise distribution $P_{\mathcal{N}}(\nu_2,\dots,\nu_N) = \prod_{n=2}^{N} \frac{\#\nu_n}{|\mathcal{D}|} \equiv \prod_{n=2}^{N} P_{\mathcal{D}}(\nu_n)$:

$$\begin{aligned}\ell(i_1,i_2,\dots,i_N) = \log\sigma([\![\mathbf{a}^{(1)}_{i_1},\mathbf{a}^{(2)}_{i_2},\dots,\mathbf{a}^{(N)}_{i_N}]\!]) \ + \\ +\ \kappa \cdot \mathop{\mathbb{E}}_{(\nu_2\dots\nu_N)\sim P_{\mathcal{N}}}\Big[\log\sigma\big(-[\![\mathbf{a}^{(1)}_{i_1},\mathbf{a}^{(2)}_{\nu_2},\dots,\mathbf{a}^{(N)}_{\nu_N}]\!]\big)\Big]\end{aligned}$$

The expectation term can be explicited:

$$\mathop{\mathbb{E}}_{(\nu_2\dots\nu_N)\sim P_{\mathcal{N}}}\Big[\log\sigma\big(-[\![\mathbf{a}^{(1)}_{i_1},\mathbf{a}^{(2)}_{\nu_2},\dots,\mathbf{a}^{(N)}_{\nu_N}]\!]\big)\Big] = \sum_{j_2\cdots j_N} P_{\mathcal{N}}(j_2,\dots,j_N)\log\sigma\big(-[\![\mathbf{a}^{(1)}_{i_1},\mathbf{a}^{(2)}_{j_2},\dots,\mathbf{a}^{(N)}_{j_N}]\!]\big)$$

Weighting the loss error for each tuple $(i_1,i_2,\dots,i_N)$ with their empirical probability $P_{\mathcal{D}}(i_1,i_2,\dots,i_N)$, and defining $[\![\mathbf{a}^{(1)}_{i_1},\mathbf{a}^{(2)}_{i_2},\dots,\mathbf{a}^{(N)}_{i_N}]\!] \equiv m_{i_1 i_2\dots i_N}$, we obtain the global objective with the sum over all combinations of vocabulary elements:

$$\begin{aligned}\mathcal{L} &= -\sum_{i_1 i_2\dots i_N} P_{\mathcal{D}}(i_1,i_2,\dots,i_N)\Big[\log\sigma(m_{i_1 i_2\dots i_N}) + \kappa\sum_{j_2\cdots j_N} P_{\mathcal{N}}(j_2,\dots,j_N)\log\sigma(-m_{i_1 j_2\dots j_N})\Big] \\ &= -\sum_{i_1 i_2\dots i_N} P_{\mathcal{D}}(i_1,i_2,\dots,i_N)\log\sigma(m_{i_1 i_2\dots i_N}) \ + \\ &\qquad -\ \kappa\sum_{i_1 i_2\dots i_N} P_{\mathcal{D}}(i_1,i_2,\dots,i_N)\sum_{j_2\cdots j_N} P_{\mathcal{N}}(j_2,\dots,j_N)\log\sigma(-m_{i_1 j_2\dots j_N})\end{aligned}$$

In the second term we can notice that only $P_{\mathcal{D}}(i_1,i_2,\dots,i_N)$ depends on the $N-1$ indices $(i_2,\dots,i_N)$, so performing the sum over that subset of indices we obtain the marginal distribution $\sum_{i_2\dots i_N} P_{\mathcal{D}}(i_1,i_2,\dots,i_N) = P_{\mathcal{D}}(i_1)$. Finally renaming indices $\{j_h\} \to \{i_h\}$ and observing that $P_{\mathcal{D}}(i_1)P_{\mathcal{N}}(i_2,\dots,i_N) \equiv P_{\mathcal{N}}(i_1,i_2,\dots,i_N)$, we obtain the final loss:

$$\mathcal{L}^{HOSGNS} = -\sum_{i_1\dots i_N}\Big[P_{\mathcal{D}}(i_1,\dots,i_N)\log\sigma(m_{i_1\dots i_N}) + \kappa\cdot P_{\mathcal{N}}(i_1,\dots,i_N)\log\sigma(-m_{i_1\dots i_N})\Big] \quad \text{(A.5)}$$

In particular for the 3rd-order and 4th-order cases, with vocabularies $\mathcal{V}_1 = \mathcal{W},\ \mathcal{V}_2 = \mathcal{C},\ \mathcal{V}_3 = \mathcal{T},\ \mathcal{V}_4 = \mathcal{S}$ and embedding matrices $\mathbf{A}^{(1)} = \mathbf{W},\ \mathbf{A}^{(2)} = \mathbf{C},\ \mathbf{A}^{(3)} = \mathbf{T},\ \mathbf{A}^{(4)} = \mathbf{S}$, we have the loss functions minimized by our time-varying graph embedding model:

$$\begin{aligned}\mathcal{L}^{(3rd)} &= -\sum_{ijk}\Big[P_{\mathcal{D}}(i,j,k)\log\sigma\big([\![\mathbf{w}_i,\mathbf{c}_j,\mathbf{t}_k]\!]\big) + \kappa\ P_{\mathcal{N}}(i,j,k)\log\sigma\big(-[\![\mathbf{w}_i,\mathbf{c}_j,\mathbf{t}_k]\!]\big)\Big] \\ \mathcal{L}^{(4th)} &= -\sum_{ijkl}\Big[P_{\mathcal{D}}(i,j,k,l)\log\sigma\big([\![\mathbf{w}_i,\mathbf{c}_j,\mathbf{t}_k,\mathbf{s}_l]\!]\big) + \kappa\ P_{\mathcal{N}}(i,j,k,l)\log\sigma\big(-[\![\mathbf{w}_i,\mathbf{c}_j,\mathbf{t}_k,\mathbf{s}_l]\!]\big)\Big] \quad \text{(A.6)}\end{aligned}$$

A.2.2 Implicit tensor factorization theorem

Here we show the equivalence of HOSGNS to tensor factorization of the shifted PMI tensor into factor matrices.

Theorem. Let $\mathcal{D} = \{(i_1, i_2, \ldots, i_N),\ i_1 \in \mathcal{V}_1,\ i_2 \in \mathcal{V}_2,\ \ldots,\ i_N \in \mathcal{V}_N\}$ a training set of higher-order co-occurrences and $\text{PMI}(i_1, \ldots, i_N) = \log\left(\frac{P_{\mathcal{D}}(i_1,\ldots,i_N)}{P_{\mathcal{N}}(i_1,\ldots,i_N)}\right)$ the entries of the pointwise mutual information tensor computed from $\mathcal{D}$. Let $\mathbf{A}^{(1)} \in \mathbb{R}^{|\mathcal{V}_1| \times d}, \ldots, \mathbf{A}^{(N)} \in \mathbb{R}^{|\mathcal{V}_N| \times d}$ embedding matrices of HOSGNS. For d sufficiently large, HOSGNS has the same global optimum as the canonical polyadic decomposition of SPMI_κ, the PMI tensor shifted by $\log \kappa$.

Proof. We consider each relation $[\![\mathbf{a}^{(1)}_{i_1}, \ldots, \mathbf{a}^{(N)}_{i_N}]\!] \equiv m_{i_1 \ldots i_N}$ as a mapping from combinations of embedding vectors to elements of a tensor $\boldsymbol{\mathcal{M}} \in \mathbb{R}^{|\mathcal{V}_1| \times \cdots \times |\mathcal{V}_N|}$. The global loss $\mathcal{L} = \sum_{i_1 \ldots i_N} \mathcal{L}(i_1, \ldots, i_N)$ in Eq. (A.5) is the sum of local losses computed from elements of $\boldsymbol{\mathcal{M}}$:

$$\mathcal{L}(i_1, \ldots, i_N) = -\left[P_{\mathcal{D}}(i_1, \ldots, i_N) \log \sigma(m_{i_1 \ldots i_N}) + \kappa\ P_{\mathcal{N}}(i_1, \ldots, i_N) \log \sigma(-m_{i_1 \ldots i_N})\right]$$

For sufficiently large d (i.e. allowing for a perfect reconstruction of SPMI_κ), each $m_{i_1 \ldots i_N}$ can assume a value independently of the others, and we can treat the loss function $\mathcal{L}$ as a sum of independent addends, restricting the optimization problem to looking at the local objective and its derivative respect to $m_{i_1 \ldots i_N}$:

$$\begin{aligned} \frac{\partial \mathcal{L}(i_1, \ldots, i_N)}{\partial m_{i_1 \ldots i_N}} &= \kappa\ P_{\mathcal{N}}(i_1, \ldots, i_N) \sigma(m_{i_1 \ldots i_N}) - P_{\mathcal{D}}(i_1, \ldots, i_N)\big[1 - \sigma(m_{i_1 \ldots i_N})\big] \\ &= \big[P_{\mathcal{D}}(i_1, \ldots, i_N) + \kappa\ P_{\mathcal{N}}(i_1, \ldots, i_N)\big] \sigma(m_{i_1 \ldots i_N}) - P_{\mathcal{D}}(i_1, \ldots, i_N) \end{aligned}$$

where we have used $\frac{d\sigma}{dx} = \sigma(x)(1 - \sigma(x))$. To compare the derivative with zero, we use the identities $P_{\mathcal{D}} = (P_{\mathcal{D}} + \kappa\ P_{\mathcal{N}})(1 + \frac{\kappa\ P_{\mathcal{N}}}{P_{\mathcal{D}}})^{-1}$ and $(1 + x)^{-1} = \sigma(\log x^{-1})$:

$$\frac{\partial \mathcal{L}(i_1, \ldots, i_N)}{\partial m_{i_1 \ldots i_N}} = [P_{\mathcal{D}}(i_1, \ldots, i_N) + \kappa\ P_{\mathcal{N}}(i_1, \ldots, i_N)] \left[\sigma(m_{i_1 \ldots i_N}) - \sigma\left(\log \frac{P_{\mathcal{D}}(i_1, \ldots, i_N)}{\kappa\ P_{\mathcal{N}}(i_1, \ldots, i_N)}\right)\right]$$

from which it follows that the derivative is 0 when elements $m_{i_1 \ldots i_N}$ are equal to the shifted PMI tensor entries:

$$\frac{\partial \mathcal{L}(i_1, \ldots, i_N)}{\partial m_{i_1 \ldots i_N}} = 0 \quad \Leftrightarrow \quad \sum_{r=1}^{d} \mathbf{A}^{(1)}_{i_1 r} \ldots \mathbf{A}^{(N)}_{i_N r} = \log\left(\frac{P_{\mathcal{D}}(i_1, \ldots, i_N)}{\kappa\ P_{\mathcal{N}}(i_1, \ldots, i_N)}\right) = \text{SPMI}_\kappa(i_1, \ldots, i_N) \tag{A.7}$$

Since we have assumed that d is large enough to ensure an exact reconstruction of SPMI_κ, and this is true if $d \approx R = \text{rank}(\text{SPMI}_\kappa)$, Eq. (A.7) is consistent with the canonical polyadic decomposition of the shifted PMI tensor. □

A.3 Description of the warm-up procedure

Warm-up is usually referred as the sequence of weights updates, at the beginning of training, performed in order to reduce over-fitting at early stages, and especially it is useful when data samples are highly differentiated [2, 3]. Here we designed the warm-up strategy with a different aim, i.e. finding an advantageous configuration of model parameters to initialize trainable weights. In particular we show that we can preliminarily optimize embedding vectors in order to ensure that all higher-order products $m_{ijk\ldots} = [\![\mathbf{w}_i, \mathbf{c}_j, \mathbf{t}_k \ldots]\!]$ return the same quantity m, regardless of the indices combination $(i, j, k \ldots)$. The value m can be chosen in order to make the cross entropy error as minimum as possible before passing empirical data samples to the model.
We start with a random initialization where embedding weights are realizations of random variables i.i.d. according to a normal distribution:

$$\mathbf{W}_{ir}, \mathbf{C}_{jr}, \mathbf{T}_{kr} \cdots \sim \mathcal{N}(0, d^{-2})\ ,\ r = 1 \ldots d$$

Once chosen m we can fix Hadamard products optimizing a squared error loss function:

$$\mathcal{L}^{(\text{warm-up})} = \sum_{ijk\ldots} \Big([\![\mathbf{w}_i, \mathbf{c}_j, \mathbf{t}_k \ldots]\!] - m\Big)^2$$

The optimal value of m is stated by the following theorem:

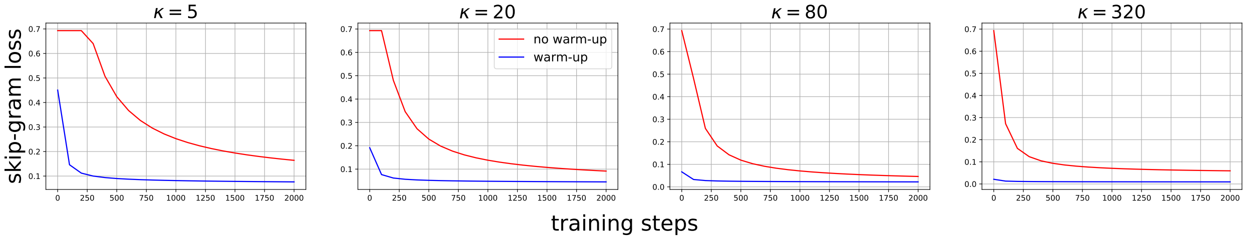

Figure 1: Impact of additional warm-up steps in the decrease of HOSGNS$^{(dyn)}$ loss function $\mathcal{L}^{(\mathrm{bce})}$ respect to the number of training iterations, for LyonSchool dataset and with different negative sampling sizes κ. The loss function is normalized with a factor $(\kappa + 1)$ for accounting the different contribute of the negative sampling parameter in $\mathcal{L}^{(\mathrm{bce})}$.

Theorem. Assuming the same value for each higher-order inner product in the set:

$$\mathcal{S} = \{m_{ijk\ldots}, i \in \mathcal{W},\ j \in \mathcal{C},\ k \in \mathcal{T}, \ldots\}$$

the cross entropy error of HOSGNS (Equation (15) of the main paper) is minimum when every $m_{ijk\ldots} \equiv m = -\log \kappa$.

Proof. Given the hypothesis, the objective function to minimize takes the form:

$$\begin{aligned}\mathcal{L}^{(\mathrm{bce})} &= -\frac{1}{B}\Big[\sum_{(ijk\ldots)\sim P_{\mathcal{D}}}^{B} \log \sigma(m) + \kappa \sum_{(ijk\ldots)\sim P_{\mathcal{N}}}^{B} \log \sigma(-m)\Big] \\ &= -\big[\log \sigma(m) + \kappa \log \sigma(-m)\big] \equiv \ell(m)\end{aligned}$$

where m is the returned value for each $m_{ijk\ldots} \in \mathcal{S}$. Solving the equation $\frac{dl}{dm} = 0$ we get:

$$\frac{1}{\sigma(m)}\frac{d\sigma}{dx}\Big|_{x=m} - \kappa \frac{1}{\sigma(-m)}\frac{d\sigma}{dx}\Big|_{x=-m} = 0 \Rightarrow \kappa = \frac{\sigma(-m)}{\sigma(m)} = e^{-m} \Rightarrow m = -\log \kappa$$

□

In Figure 1 is shown the effectiveness of the addition of extra warm-up steps in loss optimization.

A.4 Description of parameter settings

All the experiments are executed on a 64 bit Ubuntu 18.04.4 LTS system with Intel(R) Core(TM) i7-5930K CPU, 6 cores, 3.50GHz clock frequency, 64 GB RAM, and two Nvidia GeForce GTX Titan X, each with 12 GB memory. Unless otherwise declared, all the embeddings are trained with a dimension d of 128 for node classification and 192 for event-related tasks.

HOSGNS variants were optimized with Adam [4] fixing the negative samples weight $\kappa = 5$, the sample size $B = 50000$ and linearly decaying the learning rate from a starting value of 0.05 for 4000 iterations. For $\boldsymbol{\mathcal{A}}^{(dyn)}$ we set the random walks context window $T = 10$. Before training we apply 100 warm-up steps with uniform sampling of 10^5 terms per iteration in the squared loss. Models are implemented in Tensorflow[1]. The implementation of event sampling is made through the realization of a tensor with the probability distributions $\boldsymbol{\mathcal{P}}^{(stat)}$ and $\boldsymbol{\mathcal{P}}^{(dyn)}$ defined in Eq. (12) and Eq. (13). In the case of synthetic datasets, due to the huge size and low sparsity of the probabilities tensor, HOSGNS was implemented by sampling positive and negative events from a corpus of random walks. For HOSGNS$^{(stat)}$ random walks are sampled from the set of temporal snapshots $\{\mathcal{G}^{(k)}\}_{k\in\mathcal{T}}$ with window size $T = 1$, and for HOSGNS$^{(dyn)}$ random walks are sampled from the supra-adjacency graph $\mathcal{G}_{\mathcal{H}}$ with window size $T = 10$. With these sampling strategies, positive examples are drawn from the same probability distributions as in $\boldsymbol{\mathcal{P}}^{(stat)}$ and $\boldsymbol{\mathcal{P}}^{(dyn)}$. The batch size

[1] `https://github.com/tensorflow/tensorflow`

of positive examples is fixed to 20000, and for each element in the batch $\kappa = 5$ negative tuples are sampled from the corpus. Embedding parameters are initialized with 1000 warm-up steps.

For **DyANE** , as in the original paper, we optimized NODE2VEC[2] with default hyperparameters ($p = q = 1$, the same value κ=5 for negative samples and the same context window size $T = 10$ that we chose for HOSGNS). The number of SGD epochs is 1 since we did not observe any improvement in downstream tasks by increasing the number of epochs.

For **DynGEM**, with the code made available online by the authors[3], we trained the model with SGD with momentum (learning rate 10^{-3} and momentum coefficient 0.99) for 100 iterations in the first time-step and 30 for the others. We set the internal layer sizes of the autoencoder to $[400, 250, d]$.

DynamicTriad is trained with Adagrad (learning rate 10^{-1}) with 100 epochs and negative/positive samples ratio set to 5. Coefficients β_0 and β_1 related to social homofily and temporal smoothness are set to 0.1. We used the reference implementation available in the official repository[4].

DySAT is trained using the standard implementation[5] with Adam optimizer (initial learning rate 10^{-3}) for 100 epochs with window size for temporal attention set to 10, spatial and temporal drop-out probabilities equal to 0.1 and 0.5 respectively.

ISGNS is trained using the reference code[6] with standard NODE2VEC parameters (the same as DyANE). We tested a few combinations of other hyperparameters, and reported the results with the ones described above, since we observed that the improvement is minimal and does not invalidate the results. Due to the stochastic nature of the training, each of the above embedding models is trained 5 times for more robust performance estimates in downstream tasks.

A.5 Extensive results of embedding representations in downstream tasks

Here we report complete results about empirical and synthetic datasets in downstream tasks already reported (partially) in the main paper.
In Tables 2, 3, 4, 5 and 6 we report the whole set of Macro-F1 scores in node classification, event reconstruction and prediction, with different operations used to construct embeddings for the logistic regression. In Table 1 we present definitions of different operators employed (Hadamard included, the only one displayed in the paper). For node classification, we show in Tables 2, 3 and 5 results related to all tested combinations of epidemic parameters (β, μ) used to simulate SIR processes.
In Figures 2, 3 4 we report a sensitivity analysis with the effect of the embedding size d, the negative sampling constant κ and the number of training steps E on prediction performances in node classication and event-related tasks.

A.6 Impact of the aggregation time window on SIR node classification

In Figure 5 we show the prediction performance of embedding models DyANE and HOSGNS$^{(dyn)}$ in node classification task, changing the width of the aggregation window in SocioPatterns data. In HOSGNS$^{(dyn)}$ we notice a modest descent of classification performance for larger aggregation windows, while results for DyANE are more unstable, resulting in a less robust performance respect to the proposed HOSGNS model.

A.7 Comparison with canonical tensor decomposition

In Figure 6 we probe the capability of the HOSGNS models to reconstruct the shifted PMI tensor entries in empirical datasets by computing the higher order product of embedding vectors, operation optimized during the training phase to classify non-zero elements of the tensor itself. We verify the goodness of approximation estimating the square of the Pearson coefficient between the distribution of actual PMI values and the estimated ones, having fixed the model $\kappa = 5$ during training. In addition we report in Tables 7, 8 and 9 results about node classification and link-related tasks comparing HOSGNS$^{(stat)}$ and HOSGNS$^{(dyn)}$

[2]`https://github.com/snap-stanford/snap/tree/master/examples/node2vec`
[3]`http://www-scf.usc.edu/~nkamra/`
[4]`https://github.com/luckiezhou/DynamicTriad`
[5]`https://github.com/aravindsankar28/DySAT`
[6]`https://github.com/RingBDStack/dynamic_network_embedding`

Table 1: Operators and their definitions used to combine different embeddings learned with HOSGNS for tensors of order 3 ($\text{HOSGNS}^{(stat)}$) and 4 ($\text{HOSGNS}^{(dyn)}$ and $\text{HOSGNS}^{(stat|dyn)}$), applied to temporal node $i^{(k)}$ in node classification and to link (i, j, k) in temporal event reconstruction. All operations, except *Concat*, are described element-wise.

Operator	SGNS order	Node Classification	Event Reconstruction and Prediction
Average	$3rd, 4th$	$\frac{1}{2}(\mathbf{w}_i + \mathbf{t}_k)$	$\frac{1}{3}(\mathbf{w}_i + \mathbf{c}_j + \mathbf{t}_k)$
Hadamard	$3rd$<br>$4th$	$\mathbf{w}_i \circ \mathbf{t}_k$	$\mathbf{w}_i \circ \mathbf{c}_j \circ \mathbf{t}_k$<br>$\mathbf{w}_i \circ \mathbf{c}_j \circ \mathbf{t}_k \circ \mathbf{s}_k$
Weighted-L1	$3rd, 4th$	$\lvert\mathbf{w}_i - \mathbf{t}_k\rvert$	$\frac{1}{3}(\lvert\mathbf{w}_i - \mathbf{t}_k\rvert + \lvert\mathbf{w}_i - \mathbf{c}_j\rvert + \lvert\mathbf{c}_j - \mathbf{t}_k\rvert)$
Weighted-L2	$3rd, 4th$	$(\mathbf{w}_i - \mathbf{t}_k)^2$	$\frac{1}{3}[(\mathbf{w}_i - \mathbf{t}_k)^2 + (\mathbf{w}_i - \mathbf{c}_j)^2 + (\mathbf{c}_j - \mathbf{t}_k)^2]$
Concat	$3rd, 4th$	$[\mathbf{w}_i, \mathbf{t}_k]$	$[\mathbf{w}_i, \mathbf{c}_j, \mathbf{t}_k]$

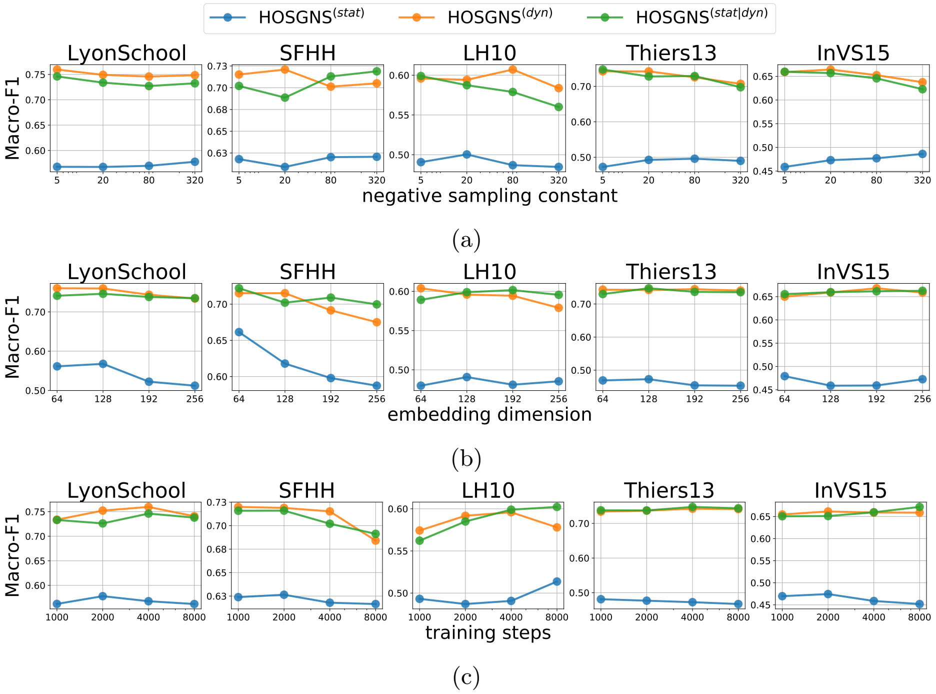

Figure 2: Macro-F1 scores related to classification of nodes in SIR states from simulations with epidemic parameters $(\beta, \mu) = (0.125, 0.001)$, computed (a) varying the negative sampling parameter κ, (b) varying the embedding dimension and (c) varying the number of training iterations E. In each panel remaining parameters are fixed to $d = 128$, $\kappa = 5$ and $E = 4000$. Time-resolved embedding vectors of nodes are computed with Hadamard product as explained in Table 1.

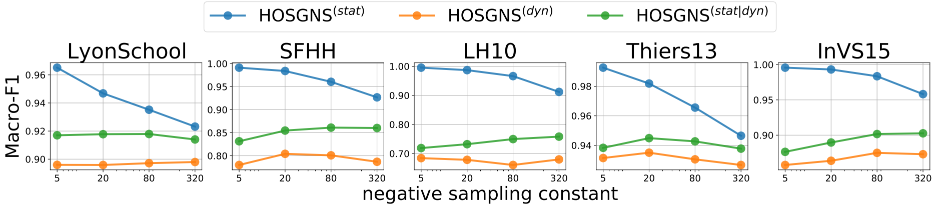

(a)

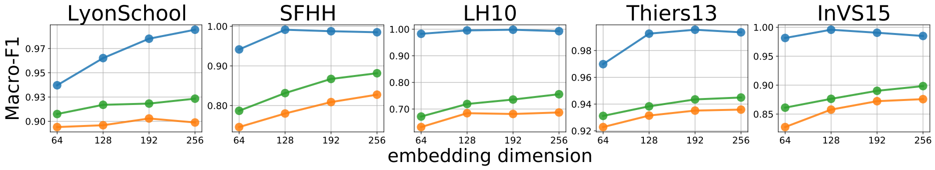

(b)

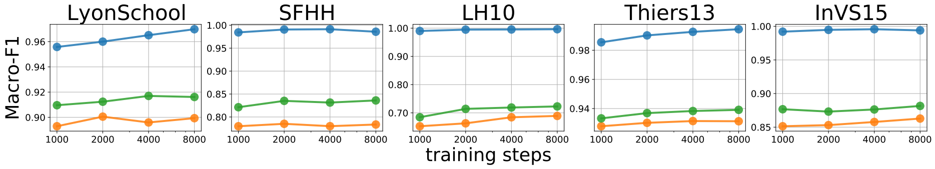

(c)

Figure 3: Macro-F1 scores related to temporal event reconstruction, computed (a) varying the negative sampling parameter κ, (b) varying the embedding dimension and (c) varying the number of training iterations E. In each panel remaining parameters are fixed to $d = 128$, $\kappa = 5$ and $E = 4000$. Time-resolved embedding vectors of edges are computed with Hadamard product as explained in Table 1.

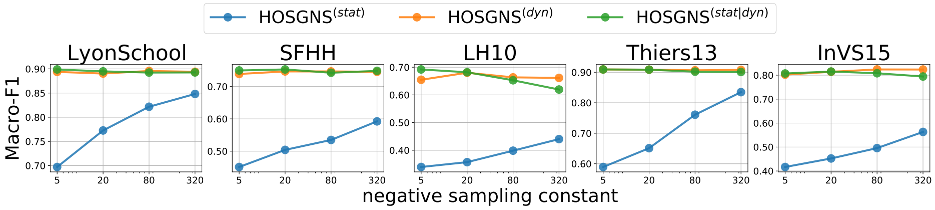

(a)

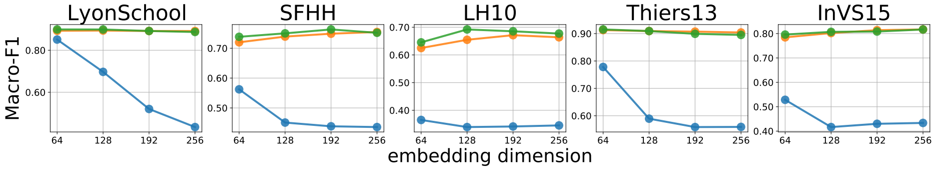

(b)

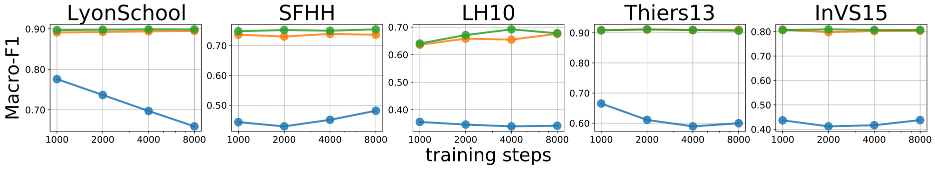

(c)

Figure 4: Macro-F1 scores related to missing event prediction, computed (a) varying the negative sampling parameter κ, (b) varying the embedding dimension and (c) varying the number of training iterations E. In each panel remaining parameters are fixed to $d = 128$, $\kappa = 5$ and $E = 4000$. Time-resolved embedding vectors of edges are computed with Hadamard product as explained in Table 1.

Table 2: Macro-F1 scores for classification of nodes in epidemic states according to different SIR processes for empirical datasets, with parameters (β, μ) previously shown in the paper. Here for each HOSGNS variant we tested different operators to produce node-time representations, all with a dimension $d = 128$, used as input to a Logistic Regression. For each (β, μ) we highlight the two highest scores and underline the best one.

(β, μ)	Model	Operator	Dataset				
			LYONSCHOOL	SFHH	LH10	THIERS13	INVS15
	DYANE		$\mathbf{78.1 \pm 0.5}$	67.0 ± 1.2	52.5 ± 1.7	71.9 ± 0.6	$\mathbf{64.3 \pm 0.8}$
	DYNGEM	-	58.7 ± 2.8	35.9 ± 1.1	34.5 ± 0.7	35.5 ± 1.2	58.8 ± 1.1
	DYNAMICTRIAD		31.0 ± 0.4	28.8 ± 0.4	29.9 ± 0.3	30.3 ± 0.2	30.4 ± 0.2
	DYSAT		27.3 ± 0.2	27.4 ± 0.3	29.7 ± 0.2	30.2 ± 0.2	30.5 ± 0.2
	ISGNS		63.5 ± 0.6	60.7 ± 0.8	54.1 ± 1.1	56.4 ± 0.6	52.3 ± 0.6
		Average	56.3 ± 0.9	55.5 ± 1.3	51.1 ± 1.1	58.0 ± 0.5	53.0 ± 0.8
		Hadamard	55.5 ± 0.8	57.3 ± 1.1	45.9 ± 0.9	46.9 ± 0.7	44.5 ± 0.7
	HOSGNS$^{(stat)}$	Weighted-L1	53.9 ± 0.9	49.2 ± 0.9	49.5 ± 1.2	46.7 ± 0.6	45.5 ± 0.8
		Weighted-L2	53.2 ± 0.7	47.5 ± 0.9	48.8 ± 1.0	46.8 ± 0.6	45.0 ± 0.7
(0.25, 0.002)		Concat	69.4 ± 0.9	59.8 ± 1.4	54.4 ± 1.2	61.2 ± 0.8	56.6 ± 0.8
		Average	73.1 ± 0.5	66.5 ± 1.1	62.5 ± 1.5	69.5 ± 0.9	62.4 ± 0.8
		Hadamard	$\mathbf{\underline{79.2} \pm 0.5}$	$\mathbf{\underline{69.1} \pm 1.1}$	59.6 ± 1.5	71.8 ± 1.2	$\mathbf{\underline{64.6} \pm 0.7}$
	HOSGNS$^{(dyn)}$	Weighted-L1	75.7 ± 0.5	66.2 ± 1.2	59.0 ± 1.0	70.8 ± 0.7	61.9 ± 0.8
		Weighted-L2	74.9 ± 0.6	67.0 ± 1.1	60.5 ± 1.2	72.2 ± 0.5	62.5 ± 0.6
		Concat	77.1 ± 0.5	$\mathbf{68.8 \pm 1.0}$	$\mathbf{\underline{63.5} \pm 1.5}$	$\mathbf{\underline{72.9} \pm 0.6}$	63.1 ± 0.8
		Average	72.3 ± 0.5	65.2 ± 0.9	61.0 ± 1.3	69.5 ± 0.7	62.5 ± 1.0
		Hadamard	77.4 ± 0.6	67.4 ± 1.2	59.7 ± 1.2	$\mathbf{72.5 \pm 0.7}$	64.2 ± 1.0
	HOSGNS$^{(stat\|dyn)}$	Weighted-L1	73.4 ± 0.8	66.7 ± 1.1	57.2 ± 1.3	70.1 ± 0.8	63.1 ± 0.9
		Weighted-L2	73.4 ± 0.6	65.0 ± 1.2	57.8 ± 1.2	70.0 ± 0.7	63.5 ± 0.9
		Concat	76.1 ± 0.6	67.9 ± 1.1	$\mathbf{62.6 \pm 1.5}$	70.9 ± 0.6	62.0 ± 0.8
	DYANE		72.2 ± 0.6	64.9 ± 1.7	59.0 ± 1.2	68.0 ± 0.5	$\mathbf{\underline{60.2} \pm 0.5}$
	DYNGEM	-	56.4 ± 2.7	35.9 ± 4.1	35.8 ± 1.2	32.9 ± 1.2	55.0 ± 0.6
	DYNAMICTRIAD		29.5 ± 0.5	33.1 ± 2.5	29.6 ± 0.4	27.4 ± 0.3	28.4 ± 0.2
	DYSAT		26.4 ± 0.2	29.5 ± 1.3	29.5 ± 0.3	26.5 ± 0.2	28.5 ± 0.2
	ISGNS		59.2 ± 0.3	57.1 ± 1.6	55.9 ± 1.0	49.0 ± 0.3	47.2 ± 0.3
		Average	54.7 ± 0.8	59.0 ± 2.6	51.2 ± 1.1	56.7 ± 0.5	52.1 ± 0.5
		Hadamard	55.5 ± 0.7	57.6 ± 2.2	49.4 ± 0.8	45.5 ± 0.4	43.6 ± 0.5
	HOSGNS$^{(stat)}$	Weighted-L1	53.7 ± 0.7	52.7 ± 2.9	49.7 ± 1.1	46.2 ± 0.5	43.9 ± 0.5
		Weighted-L2	54.0 ± 0.8	51.4 ± 3.0	48.0 ± 0.9	45.5 ± 0.4	43.0 ± 0.5
(0.0625, 0.002)		Concat	65.5 ± 0.7	61.3 ± 2.6	55.3 ± 1.4	61.9 ± 0.5	53.3 ± 0.6
		Average	67.8 ± 0.7	66.6 ± 2.1	$\mathbf{63.0 \pm 1.2}$	67.6 ± 0.4	59.1 ± 0.6
		Hadamard	$\mathbf{\underline{73.5} \pm 0.5}$	65.7 ± 1.6	61.1 ± 1.2	$\mathbf{\underline{69.5} \pm 0.3}$	59.6 ± 0.5
	HOSGNS$^{(dyn)}$	Weighted-L1	70.8 ± 0.6	65.9 ± 1.9	62.4 ± 1.3	67.5 ± 0.4	58.0 ± 0.6
		Weighted-L2	71.4 ± 0.5	66.5 ± 2.2	59.0 ± 1.1	68.5 ± 0.5	57.5 ± 0.7
		Concat	72.0 ± 0.7	$\mathbf{\underline{68.3} \pm 2.1}$	$\mathbf{\underline{64.0} \pm 1.5}$	68.2 ± 0.4	59.9 ± 0.6
		Average	67.8 ± 0.5	64.3 ± 1.9	60.1 ± 1.2	66.7 ± 0.4	$\mathbf{\underline{60.2} \pm 0.5}$
		Hadamard	$\mathbf{72.9 \pm 0.6}$	66.3 ± 1.9	58.2 ± 1.1	$\mathbf{68.5 \pm 0.4}$	59.0 ± 0.7
	HOSGNS$^{(stat\|dyn)}$	Weighted-L1	70.3 ± 0.5	$\mathbf{67.2 \pm 2.2}$	59.2 ± 1.2	67.2 ± 0.5	58.0 ± 0.5
		Weighted-L2	70.0 ± 0.5	66.6 ± 2.2	59.5 ± 1.3	66.7 ± 0.5	57.6 ± 0.6
		Concat	71.3 ± 0.6	66.8 ± 2.2	62.8 ± 1.6	68.3 ± 0.3	59.2 ± 0.6
	DYANE		$\mathbf{74.7 \pm 0.7}$	67.7 ± 1.2	$\mathbf{\underline{63.4} \pm 1.8}$	72.7 ± 0.4	$\mathbf{\underline{68.6} \pm 0.4}$
	DYNGEM	-	57.4 ± 2.8	36.2 ± 2.6	41.4 ± 1.3	34.8 ± 1.3	61.2 ± 0.9
	DYNAMICTRIAD		32.3 ± 0.5	31.5 ± 0.8	30.5 ± 0.4	27.9 ± 0.3	30.0 ± 0.2
	DYSAT		26.4 ± 0.2	29.4 ± 0.8	30.0 ± 0.3	27.7 ± 0.3	29.9 ± 0.2
	ISGNS		65.1 ± 0.5	63.0 ± 1.4	60.2 ± 1.7	56.0 ± 0.5	52.5 ± 0.5
		Average	56.4 ± 0.8	57.6 ± 1.7	50.5 ± 1.4	58.4 ± 0.8	56.9 ± 0.8
		Hadamard	56.9 ± 0.8	59.4 ± 1.7	48.5 ± 1.1	49.0 ± 0.6	46.2 ± 0.8
	HOSGNS$^{(stat)}$	Weighted-L1	53.5 ± 0.9	51.3 ± 1.8	47.3 ± 0.8	48.2 ± 0.5	47.7 ± 0.6
		Weighted-L2	52.4 ± 0.9	48.9 ± 1.9	47.1 ± 1.1	48.5 ± 0.6	47.2 ± 0.7
(0.1875, 0.001)		Concat	67.3 ± 0.7	62.7 ± 1.5	51.6 ± 1.6	63.7 ± 0.8	59.5 ± 0.8
		Average	69.9 ± 0.5	66.3 ± 1.5	$\mathbf{62.6 \pm 1.9}$	71.0 ± 0.7	65.4 ± 0.8
		Hadamard	$\mathbf{\underline{76.5} \pm 0.4}$	68.6 ± 1.1	62.4 ± 1.7	$\mathbf{\underline{74.8} \pm 0.5}$	$\mathbf{67.9 \pm 0.7}$
	HOSGNS$^{(dyn)}$	Weighted-L1	72.1 ± 0.5	68.3 ± 1.5	62.4 ± 1.9	72.5 ± 0.6	64.9 ± 0.7
		Weighted-L2	71.5 ± 0.5	67.7 ± 1.4	60.7 ± 1.9	72.5 ± 0.6	66.4 ± 0.7
		Concat	73.0 ± 0.5	$\mathbf{69.1 \pm 1.4}$	59.6 ± 2.1	72.9 ± 0.6	65.1 ± 0.7
		Average	69.6 ± 0.7	66.2 ± 1.4	61.6 ± 1.8	69.6 ± 0.7	66.1 ± 0.6
		Hadamard	74.5 ± 0.4	$\mathbf{\underline{69.4} \pm 1.4}$	62.5 ± 2.0	$\mathbf{73.6 \pm 0.6}$	67.3 ± 0.5
	HOSGNS$^{(stat\|dyn)}$	Weighted-L1	71.1 ± 0.6	68.5 ± 1.4	58.6 ± 1.8	71.0 ± 0.6	66.0 ± 0.8
		Weighted-L2	71.0 ± 0.6	67.1 ± 1.5	59.0 ± 1.6	71.0 ± 0.7	65.8 ± 0.5
		Concat	73.0 ± 0.6	68.2 ± 1.4	60.1 ± 1.8	72.1 ± 0.7	65.1 ± 0.8

Table 3: Macro-F1 scores for classification of nodes in epidemic states according to different SIR processes for empirical datasets, with parameters (β, μ) not shown in the paper. Here for each HOSGNS variant we tested different operators to produce node-time representations, all with a dimension $d = 128$, used as input to a Logistic Regression. For each (β, μ) we highlight the two highest scores and underline the best one. In the case $(\beta, \mu) = (0.125, 0.004)$ results for datasets LH10 and InVS15 are discarded since the SIR simulation does not meet the condition $|I_{|\mathcal{T}|/2}| \geq 1$, as explained in DyANE.

(β, μ)	Model	Operator	Dataset<br>LYONSCHOOL	 SFHH	 LH10	 THIERS13	 INVS15
(0.125, 0.002)	DYANE		**77.1 ± 0.4**	**68.4 ± 0.9**	54.8 ± 1.5	71.6 ± 0.4	62.4 ± 0.5
	DYNGEM	-	57.1 ± 2.7	32.8 ± 1.3	35.0 ± 0.8	34.4 ± 1.0	57.1 ± 0.7
	DYNAMICTRIAD		30.4 ± 0.4	29.3 ± 0.4	30.1 ± 0.3	29.0 ± 0.3	29.4 ± 0.2
	DYSAT		27.0 ± 0.1	27.1 ± 0.3	30.3 ± 0.3	28.3 ± 0.3	29.4 ± 0.2
	ISGNS		61.8 ± 0.4	58.2 ± 0.7	54.6 ± 1.2	51.4 ± 0.3	49.1 ± 0.4
	HOSGNS$^{(stat)}$	Average	57.5 ± 0.8	54.0 ± 0.9	48.9 ± 1.0	58.0 ± 0.7	53.7 ± 0.6
		Hadamard	55.4 ± 0.9	55.9 ± 0.8	44.9 ± 1.0	46.3 ± 0.4	44.8 ± 0.6
		Weighted-L1	53.8 ± 0.8	48.5 ± 0.8	48.2 ± 1.0	46.9 ± 0.5	45.8 ± 0.6
		Weighted-L2	52.6 ± 0.8	46.5 ± 0.8	46.9 ± 1.0	45.2 ± 0.5	44.0 ± 0.7
		Concat	69.5 ± 0.5	59.0 ± 1.0	53.8 ± 1.3	62.1 ± 0.8	56.2 ± 0.6
	HOSGNS$^{(dyn)}$	Average	72.1 ± 0.6	66.0 ± 0.9	**60.8 ± 1.2**	70.1 ± 0.5	61.2 ± 0.7
		Hadamard	**77.5 ± 0.5**	**68.8 ± 0.8**	58.7 ± 1.1	**72.6 ± 0.5**	**63.3 ± 0.6**
		Weighted-L1	73.5 ± 0.6	66.4 ± 0.8	59.6 ± 1.5	70.7 ± 0.5	60.2 ± 0.5
		Weighted-L2	73.8 ± 0.5	66.1 ± 1.0	58.6 ± 1.2	71.0 ± 0.4	61.0 ± 0.7
		Concat	74.8 ± 0.5	67.5 ± 0.8	**62.2 ± 1.5**	71.0 ± 0.4	62.9 ± 0.5
	HOSGNS$^{(stat\|dyn)}$	Average	71.2 ± 0.8	65.8 ± 0.8	59.4 ± 1.0	69.6 ± 0.5	62.0 ± 0.6
		Hadamard	75.2 ± 0.6	68.1 ± 0.8	59.7 ± 1.1	**72.0 ± 0.5**	**63.4 ± 0.6**
		Weighted-L1	73.0 ± 0.5	64.7 ± 0.8	57.3 ± 1.2	70.0 ± 0.5	61.0 ± 0.6
		Weighted-L2	72.0 ± 0.6	63.8 ± 0.9	57.0 ± 0.9	70.1 ± 0.6	62.4 ± 0.6
		Concat	73.7 ± 0.6	66.5 ± 0.8	60.1 ± 1.3	70.3 ± 0.5	61.6 ± 0.8
(0.125, 0.001)	DYANE		**75.3 ± 0.4**	**71.6 ± 1.9**	59.0 ± 1.8	72.4 ± 0.3	65.8 ± 0.6
	DYNGEM	-	58.9 ± 2.9	37.0 ± 4.1	41.0 ± 1.4	32.5 ± 1.2	59.0 ± 1.2
	DYNAMICTRIAD		31.2 ± 0.5	35.0 ± 3.3	30.5 ± 0.7	27.4 ± 0.3	29.5 ± 0.2
	DYSAT		25.9 ± 0.2	30.4 ± 1.2	30.3 ± 0.7	26.9 ± 0.2	29.3 ± 0.2
	ISGNS		65.5 ± 0.5	59.4 ± 0.8	57.9 ± 1.3	54.0 ± 0.4	50.6 ± 0.4
	HOSGNS$^{(stat)}$	Average	54.9 ± 0.9	59.4 ± 2.6	50.4 ± 2.1	59.8 ± 0.5	55.5 ± 0.6
		Hadamard	56.8 ± 0.9	61.8 ± 2.4	49.1 ± 1.9	47.3 ± 0.6	45.9 ± 0.7
		Weighted-L1	55.5 ± 0.7	54.5 ± 2.9	49.7 ± 2.0	49.8 ± 0.6	46.8 ± 0.6
		Weighted-L2	52.6 ± 0.8	53.0 ± 3.0	47.9 ± 2.1	47.7 ± 0.5	45.3 ± 0.6
		Concat	66.6 ± 1.2	65.9 ± 2.2	52.2 ± 1.9	63.8 ± 0.5	58.4 ± 0.6
	HOSGNS$^{(dyn)}$	Average	68.0 ± 1.2	68.5 ± 2.1	59.0 ± 2.1	71.0 ± 0.7	65.2 ± 0.7
		Hadamard	**76.0 ± 0.4**	**71.5 ± 2.0**	59.6 ± 2.0	**74.2 ± 0.4**	**65.9 ± 0.6**
		Weighted-L1	73.2 ± 0.5	69.2 ± 2.0	58.7 ± 1.7	72.6 ± 0.5	65.6 ± 0.5
		Weighted-L2	71.2 ± 0.7	69.4 ± 2.0	59.1 ± 2.2	73.2 ± 0.4	65.2 ± 0.5
		Concat	73.1 ± 0.5	71.3 ± 1.9	57.3 ± 2.0	72.7 ± 0.5	65.5 ± 0.6
	HOSGNS$^{(stat\|dyn)}$	Average	68.0 ± 0.7	68.5 ± 2.1	58.8 ± 2.0	70.7 ± 0.5	64.6 ± 0.4
		Hadamard	74.6 ± 0.4	70.2 ± 1.9	**59.9 ± 2.3**	**74.8 ± 0.4**	**66.0 ± 0.6**
		Weighted-L1	71.8 ± 0.5	69.7 ± 2.1	58.8 ± 2.3	72.2 ± 0.5	64.8 ± 0.5
		Weighted-L2	70.8 ± 0.6	69.9 ± 2.0	58.4 ± 2.2	72.6 ± 0.5	64.7 ± 0.5
		Concat	71.8 ± 0.6	70.7 ± 1.9	**59.7 ± 2.3**	72.1 ± 0.5	65.2 ± 0.7
(0.125, 0.004)	DYANE		76.0 ± 0.5	63.0 ± 0.9		67.7 ± 0.6	
	DYNGEM	-	57.9 ± 2.5	34.0 ± 0.8	-	35.0 ± 1.1	-
	DYNAMICTRIAD		31.2 ± 0.3	29.7 ± 0.4		29.5 ± 0.2	
	DYSAT		28.7 ± 0.1	28.4 ± 0.2		29.6 ± 0.2	
	ISGNS		60.8 ± 0.7	53.3 ± 0.8		50.3 ± 0.3	
	HOSGNS$^{(stat)}$	Average	56.5 ± 0.6	52.6 ± 0.8		54.2 ± 0.7	
		Hadamard	54.5 ± 0.8	54.2 ± 0.8		44.3 ± 0.5	
		Weighted-L1	54.0 ± 0.8	46.9 ± 0.8	-	44.7 ± 0.6	-
		Weighted-L2	52.0 ± 0.9	45.6 ± 0.7		44.2 ± 0.6	
		Concat	68.2 ± 1.2	57.4 ± 1.1		58.1 ± 0.9	
	HOSGNS$^{(dyn)}$	Average	73.3 ± 0.6	62.9 ± 0.9		66.0 ± 0.7	
		Hadamard	**77.2 ± 0.4**	63.5 ± 1.0		**68.6 ± 0.6**	
		Weighted-L1	75.0 ± 0.6	62.6 ± 0.8	-	67.1 ± 0.5	-
		Weighted-L2	73.2 ± 1.2	62.3 ± 0.9		67.2 ± 0.5	
		Concat	**77.0 ± 0.4**	**64.8 ± 0.8**		67.8 ± 0.7	
	HOSGNS$^{(stat\|dyn)}$	Average	72.0 ± 0.6	60.3 ± 0.8		65.6 ± 0.6	
		Hadamard	74.4 ± 0.7	**64.5 ± 1.0**		**68.1 ± 0.6**	
		Weighted-L1	72.3 ± 0.7	61.1 ± 0.9	-	65.4 ± 0.4	-
		Weighted-L2	72.8 ± 0.6	60.3 ± 0.8		66.6 ± 0.5	
		Concat	75.2 ± 0.4	63.1 ± 1.0		67.4 ± 0.6	

Table 4: Macro-F1 scores for temporal event reconstruction (top) and missing event prediction (bottom) over empirical datasets. Here for each HOSGNS variant we tested different operators to produce link-time representations, all with a dimension $d = 192$, used as input to a Logistic Regression. We highlight in bold the best two overall scores for each dataset. For baseline models we underline their highest score.

Model	Operator	Dataset LYONSCHOOL	SFHH	LH10	THIERS13	INVS15
DYANE	Average	56.4 ± 0.4	52.9 ± 0.5	52.3 ± 0.6	51.0 ± 0.4	52.7 ± 0.4
	Hadamard	89.7 ± 0.3	86.5 ± 0.3	74.6 ± 0.6	94.7 ± 0.1	94.1 ± 0.1
	Weighted-L1	90.2 ± 0.2	83.3 ± 0.5	73.3 ± 0.7	94.7 ± 0.1	94.4 ± 0.2
	Weighted-L2	90.6 ± 0.2	84.5 ± 0.5	72.0 ± 0.5	95.0 ± 0.1	94.8 ± 0.2
	Concat	65.7 ± 0.4	53.8 ± 0.4	56.2 ± 0.6	57.0 ± 0.4	50.9 ± 0.4
DYNGEM	Average	57.7 ± 0.5	56.8 ± 0.7	54.8 ± 1.5	40.4 ± 1.5	42.8 ± 0.9
	Hadamard	62.2 ± 0.4	55.1 ± 1.0	52.5 ± 1.6	40.8 ± 1.5	43.7 ± 1.0
	Weighted-L1	58.4 ± 0.6	52.3 ± 0.7	50.9 ± 1.2	41.3 ± 1.6	44.8 ± 0.9
	Weighted-L2	53.7 ± 0.6	47.0 ± 0.8	47.0 ± 1.3	39.2 ± 1.2	43.6 ± 0.6
	Concat	60.4 ± 0.4	57.8 ± 0.3	48.9 ± 1.7	36.9 ± 1.3	45.7 ± 1.0
DYNAMICTRIAD	Average	51.7 ± 0.2	56.9 ± 0.4	60.2 ± 0.6	58.1 ± 0.2	56.1 ± 0.3
	Hadamard	60.3 ± 0.3	58.9 ± 0.4	59.5 ± 0.5	62.2 ± 0.3	64.7 ± 0.3
	Weighted-L1	79.1 ± 0.4	72.3 ± 0.4	75.5 ± 0.6	70.8 ± 0.3	78.1 ± 0.2
	Weighted-L2	77.4 ± 0.4	73.4 ± 0.4	77.4 ± 0.5	72.4 ± 0.2	78.9 ± 0.3
	Concat	52.2 ± 0.2	53.4 ± 0.3	55.9 ± 0.7	55.1 ± 0.2	53.2 ± 0.3
DYSAT	Average	51.1 ± 0.3	49.6 ± 0.4	51.6 ± 0.5	50.4 ± 0.2	50.1 ± 0.3
	Hadamard	75.1 ± 0.5	52.9 ± 0.3	54.8 ± 0.6	71.1 ± 0.4	66.8 ± 0.5
	Weighted-L1	72.4 ± 0.5	51.5 ± 0.3	56.1 ± 0.6	66.4 ± 0.4	64.8 ± 0.3
	Weighted-L2	72.4 ± 0.5	51.7 ± 0.3	56.8 ± 0.7	66.5 ± 0.4	63.7 ± 0.4
	Concat	50.0 ± 0.3	50.1 ± 0.4	52.3 ± 0.5	49.8 ± 0.2	50.9 ± 0.3
ISGNS	Average	53.4 ± 0.4	50.3 ± 0.5	48.1 ± 0.6	49.4 ± 0.4	45.9 ± 0.5
	Hadamard	90.1 ± 0.3	87.2 ± 0.4	80.8 ± 0.7	96.7 ± 0.2	96.7 ± 0.2
	Weighted-L1	89.9 ± 0.3	87.7 ± 0.4	81.6 ± 0.4	96.8 ± 0.2	96.4 ± 0.2
	Weighted-L2	89.7 ± 0.3	**88.2 ± 0.4**	**81.7 ± 0.5**	**96.9 ± 0.1**	**96.8 ± 0.2**
	Concat	57.1 ± 0.5	50.2 ± 0.4	48.8 ± 0.7	52.7 ± 0.4	43.8 ± 0.4
HOSGNS$^{(stat)}$	Average	61.2 ± 0.4	53.2 ± 0.4	53.0 ± 0.6	56.0 ± 0.4	51.3 ± 0.4
	Hadamard	**98.5 ± 0.1**	**98.8 ± 0.1**	**99.8 ± 0.1**	**99.6 ± 0.1**	**99.1 ± 0.1**
	Weighted-L1	67.2 ± 0.4	60.6 ± 0.5	57.4 ± 0.6	66.7 ± 0.6	59.7 ± 0.4
	Weighted-L2	68.5 ± 0.3	60.6 ± 0.5	55.6 ± 0.6	68.0 ± 0.4	58.8 ± 0.5
	Concat	63.3 ± 0.5	54.5 ± 0.4	52.2 ± 1.0	58.7 ± 0.7	50.5 ± 0.5
HOSGNS$^{(dyn)}$	Average	63.4 ± 0.4	53.9 ± 0.4	50.3 ± 0.9	57.2 ± 0.5	50.7 ± 0.5
	Hadamard	90.3 ± 0.2	80.9 ± 0.4	68.1 ± 0.7	93.5 ± 0.2	87.2 ± 0.2
	Weighted-L1	80.5 ± 0.4	63.2 ± 0.4	56.6 ± 0.9	82.1 ± 0.4	66.5 ± 0.4
	Weighted-L2	80.4 ± 0.4	63.7 ± 0.4	56.4 ± 0.6	82.1 ± 0.3	62.9 ± 0.4
	Concat	64.1 ± 0.4	53.9 ± 0.4	50.9 ± 0.9	58.2 ± 0.7	50.9 ± 0.5
HOSGNS$^{(stat\|dyn)}$	Average	63.4 ± 0.4	54.2 ± 0.5	52.6 ± 0.8	56.8 ± 0.6	50.4 ± 0.5
	Hadamard	**91.8 ± 0.2**	86.7 ± 0.4	73.6 ± 0.6	94.3 ± 0.1	89.0 ± 0.2
	Weighted-L1	81.5 ± 0.3	64.0 ± 0.4	58.1 ± 0.8	83.7 ± 0.3	66.8 ± 0.5
	Weighted-L2	81.2 ± 0.3	64.6 ± 0.5	55.4 ± 0.6	82.7 ± 0.4	63.5 ± 0.3
	Concat	61.5 ± 0.4	53.0 ± 0.4	52.9 ± 0.9	58.3 ± 0.6	49.5 ± 0.6

Model	Operator	Dataset LYONSCHOOL	SFHH	LH10	THIERS13	INVS15
DYANE	Average	56.8 ± 0.6	50.6 ± 0.8	51.3 ± 1.0	49.1 ± 0.6	49.3 ± 0.8
	Hadamard	87.3 ± 0.3	73.5 ± 0.6	67.0 ± 1.0	87.2 ± 0.3	80.1 ± 0.8
	Weighted-L1	87.8 ± 0.3	73.3 ± 0.6	65.9 ± 1.0	84.0 ± 0.4	78.4 ± 0.6
	Weighted-L2	88.5 ± 0.2	73.7 ± 0.5	66.1 ± 1.0	84.4 ± 0.4	78.9 ± 0.6
	Concat	64.4 ± 0.5	52.4 ± 0.8	51.9 ± 1.0	57.0 ± 0.6	51.4 ± 0.7
DYNGEM	Average	56.2 ± 0.5	51.8 ± 0.8	52.0 ± 1.1	49.7 ± 0.5	50.9 ± 0.7
	Hadamard	54.8 ± 0.6	51.3 ± 0.7	51.7 ± 1.2	44.7 ± 0.7	50.9 ± 0.6
	Weighted-L1	55.5 ± 0.4	48.5 ± 0.8	50.2 ± 1.0	52.2 ± 0.4	49.8 ± 0.7
	Weighted-L2	53.2 ± 0.7	47.8 ± 0.9	48.0 ± 1.1	48.9 ± 0.6	45.3 ± 0.6
	Concat	58.2 ± 0.5	50.4 ± 0.8	46.4 ± 1.4	48.8 ± 0.5	49.9 ± 0.6
DYNAMICTRIAD	Average	51.4 ± 0.4	52.6 ± 0.6	53.0 ± 0.8	52.0 ± 0.4	49.9 ± 0.7
	Hadamard	53.1 ± 0.4	49.5 ± 0.6	52.0 ± 0.8	51.7 ± 0.5	49.8 ± 0.6
	Weighted-L1	64.3 ± 0.4	56.6 ± 0.7	54.2 ± 0.9	53.6 ± 0.4	47.2 ± 0.6
	Weighted-L2	64.5 ± 0.4	57.3 ± 0.7	54.9 ± 0.9	54.5 ± 0.5	47.0 ± 0.6
	Concat	52.6 ± 0.3	51.8 ± 0.5	52.7 ± 0.9	51.5 ± 0.3	49.9 ± 0.6
DYSAT	Average	51.3 ± 0.4	51.6 ± 0.6	52.5 ± 0.8	50.0 ± 0.4	50.3 ± 0.6
	Hadamard	73.8 ± 0.6	52.5 ± 0.7	56.6 ± 0.7	68.5 ± 0.5	61.5 ± 0.8
	Weighted-L1	71.3 ± 0.5	52.0 ± 0.6	57.6 ± 0.8	63.2 ± 0.6	64.4 ± 0.5
	Weighted-L2	70.7 ± 0.5	51.5 ± 0.7	56.5 ± 0.8	63.1 ± 0.5	63.4 ± 0.5
	Concat	49.2 ± 0.4	48.8 ± 0.8	52.4 ± 0.9	49.8 ± 0.5	50.4 ± 0.6
ISGNS	Average	52.4 ± 0.6	49.5 ± 0.8	44.9 ± 0.9	48.0 ± 0.4	42.7 ± 0.8
	Hadamard	79.8 ± 0.4	59.3 ± 0.7	61.1 ± 1.2	59.3 ± 0.6	51.7 ± 0.7
	Weighted-L1	80.8 ± 0.3	59.8 ± 0.7	61.7 ± 1.0	59.0 ± 0.6	49.8 ± 0.7
	Weighted-L2	81.5 ± 0.3	60.2 ± 0.7	62.5 ± 0.9	59.9 ± 0.6	51.5 ± 0.7
	Concat	55.8 ± 0.7	50.8 ± 0.6	46.8 ± 0.8	52.2 ± 0.5	48.5 ± 0.6
HOSGNS$^{(stat)}$	Average	58.3 ± 0.5	54.9 ± 0.6	51.4 ± 1.1	57.0 ± 0.6	49.7 ± 0.7
	Hadamard	52.1 ± 0.4	43.8 ± 0.6	34.2 ± 0.2	55.9 ± 0.6	43.0 ± 0.5
	Weighted-L1	66.3 ± 0.4	57.5 ± 0.6	56.1 ± 1.0	64.1 ± 0.5	55.2 ± 0.8
	Weighted-L2	67.8 ± 0.4	58.1 ± 0.6	55.2 ± 0.9	65.3 ± 0.5	55.2 ± 0.8
	Concat	61.6 ± 0.6	54.6 ± 0.8	52.3 ± 1.2	59.3 ± 0.6	48.8 ± 0.7
HOSGNS$^{(dyn)}$	Average	62.8 ± 0.6	54.3 ± 0.7	50.3 ± 1.0	59.0 ± 0.9	49.0 ± 0.7
	Hadamard	**89.2 ± 0.2**	**74.9 ± 0.6**	**67.1 ± 0.8**	**90.7 ± 0.3**	**81.4 ± 0.5**
	Weighted-L1	80.0 ± 0.5	62.2 ± 0.7	55.8 ± 1.3	80.6 ± 0.5	61.7 ± 0.7
	Weighted-L2	79.2 ± 0.5	62.0 ± 0.8	55.2 ± 1.0	80.4 ± 0.6	58.7 ± 0.6
	Concat	63.0 ± 0.6	53.9 ± 0.8	48.4 ± 1.7	60.1 ± 0.8	48.9 ± 0.7
HOSGNS$^{(stat\|dyn)}$	Average	62.8 ± 0.7	53.8 ± 0.8	50.9 ± 1.3	59.0 ± 0.7	47.7 ± 0.7
	Hadamard	**89.2 ± 0.3**	**76.3 ± 0.7**	**68.5 ± 1.0**	**89.9 ± 0.3**	**80.8 ± 0.6**
	Weighted-L1	81.0 ± 0.4	61.4 ± 0.7	56.3 ± 1.1	81.3 ± 0.5	61.0 ± 0.8
	Weighted-L2	80.5 ± 0.3	61.9 ± 0.7	53.8 ± 1.1	80.8 ± 0.5	57.2 ± 0.8
	Concat	62.0 ± 0.7	53.2 ± 0.8	49.5 ± 1.4	59.9 ± 0.7	48.3 ± 0.8

Table 5: Macro-F1 scores for classification of nodes in epidemic states according to different SIR epidemic processes for synthetic datasets. Here for each HOSGNS variant we tested different operators to produce node-time representations, all with a dimension $d = 128$, used as input to a Logistic Regression. For each (β, μ) we highlight the two highest scores and underline the best one.

(β, μ)	Model	Operator	Dataset OPENABM-2k-100	Dataset OPENABM-5k-20
(0.25, 0.002)	DYANE	-	57.9 ± 1.8	59.6 ± 1.7
	HOSGNS$^{(stat)}$	Average	31.2 ± 0.1	27.9 ± 0.5
		Hadamard	31.2 ± 0.1	27.8 ± 0.6
		Weighted-L1	31.1 ± 0.1	28.1 ± 0.9
		Weighted-L2	31.3 ± 0.2	27.6 ± 0.6
		Concat	32.4 ± 1.1	27.8 ± 0.9
	HOSGNS$^{(dyn)}$	Average	**61.3 ± 1.3**	**60.6 ± 1.3**
		Hadamard	57.5 ± 1.8	**61.0 ± 1.1**
		Weighted-L1	56.5 ± 1.8	56.5 ± 1.9
		Weighted-L2	**60.3 ± 1.3**	55.8 ± 2.3
		Concat	49.2 ± 2.0	56.7 ± 1.8
(0.125, 0.001)	DYANE	-	**61.6 ± 1.2**	**60.6 ± 0.7**
	HOSGNS$^{(stat)}$	Average	31.5 ± 0.2	24.6 ± 1.3
		Hadamard	31.5 ± 0.2	24.8 ± 1.3
		Weighted-L1	31.5 ± 0.2	25.1 ± 1.1
		Weighted-L2	31.4 ± 0.2	23.8 ± 1.3
		Concat	30.9 ± 1.0	27.6 ± 1.7
	HOSGNS$^{(dyn)}$	Average	60.3 ± 1.5	60.3 ± 0.8
		Hadamard	61.3 ± 1.0	60.1 ± 1.1
		Weighted-L1	**62.9 ± 0.3**	55.1 ± 2.3
		Weighted-L2	60.0 ± 1.4	55.3 ± 2.2
		Concat	60.0 ± 1.1	**60.4 ± 1.1**
(0.0625, 0.002)	DYANE	-	**61.8 ± 0.4**	53.8 ± 1.3
	HOSGNS$^{(stat)}$	Average	29.9 ± 0.2	30.1 ± 1.4
		Hadamard	29.8 ± 0.2	29.4 ± 1.4
		Weighted-L1	29.8 ± 0.3	29.6 ± 0.9
		Weighted-L2	30.0 ± 0.2	30.3 ± 1.1
		Concat	30.8 ± 1.0	32.1 ± 1.9
	HOSGNS$^{(dyn)}$	Average	**61.4 ± 0.5**	**57.4 ± 1.9**
		Hadamard	59.5 ± 0.9	**54.5 ± 1.4**
		Weighted-L1	60.2 ± 1.0	51.5 ± 2.4
		Weighted-L2	61.3 ± 0.3	46.4 ± 1.9
		Concat	60.7 ± 0.5	54.3 ± 2.1
(0.125, 0.002)	DYANE	-	60.7 ± 1.1	**61.3 ± 0.6**
	HOSGNS$^{(stat)}$	Average	30.8 ± 0.2	26.6 ± 1.2
		Hadamard	30.8 ± 0.1	27.4 ± 1.2
		Weighted-L1	30.5 ± 0.2	25.1 ± 1.3
		Weighted-L2	31.0 ± 0.2	24.6 ± 1.2
		Concat	31.3 ± 1.1	27.1 ± 1.8
	HOSGNS$^{(dyn)}$	Average	61.3 ± 0.9	**61.7 ± 0.7**
		Hadamard	58.9 ± 1.4	60.7 ± 0.6
		Weighted-L1	**62.1 ± 0.5**	56.2 ± 2.4
		Weighted-L2	**61.7 ± 0.5**	54.3 ± 2.1
		Concat	58.7 ± 0.9	59.4 ± 1.3
(0.1875, 0.001)	DYANE	-	**60.3 ± 1.4**	59.6 ± 1.5
	HOSGNS$^{(stat)}$	Average	32.0 ± 0.2	25.2 ± 1.0
		Hadamard	31.9 ± 0.2	27.4 ± 0.7
		Weighted-L1	31.9 ± 0.2	26.6 ± 0.8
		Weighted-L2	31.9 ± 0.2	26.0 ± 0.7
		Concat	30.2 ± 0.4	30.8 ± 1.4
	HOSGNS$^{(dyn)}$	Average	58.8 ± 1.6	**61.6 ± 1.2**
		Hadamard	**60.5 ± 1.1**	**60.9 ± 1.0**
		Weighted-L1	59.5 ± 1.7	57.4 ± 1.9
		Weighted-L2	59.3 ± 1.7	56.5 ± 2.2
		Concat	54.5 ± 1.8	59.9 ± 1.0
(0.125, 0.004)	DYANE	-	**60.0 ± 1.1**	**60.8 ± 0.6**
	HOSGNS$^{(stat)}$	Average	29.7 ± 0.2	25.8 ± 1.1
		Hadamard	29.5 ± 0.2	27.0 ± 1.2
		Weighted-L1	29.6 ± 0.2	26.0 ± 0.8
		Weighted-L2	29.9 ± 0.2	23.7 ± 1.2
		Concat	30.8 ± 0.7	30.0 ± 1.6
	HOSGNS$^{(dyn)}$	Average	58.6 ± 1.6	59.5 ± 1.1
		Hadamard	58.4 ± 1.2	**60.1 ± 0.7**
		Weighted-L1	**60.8 ± 1.0**	56.2 ± 1.9
		Weighted-L2	59.2 ± 1.2	52.1 ± 2.6
		Concat	58.0 ± 1.3	59.2 ± 1.1

Table 6: Macro-F1 scores in temporal event reconstruction and missing event prediction for synthetic datasets. Here for each HOSGNS variant we tested different operators to produce link-time representations, all with a dimension $d = 192$, used as input to a Logistic Regression. We highlight in bold the best two overall scores for each dataset. For baseline models we underline their highest score.

Model	Operator	OPENABM-2k-100 Reconstruction	OPENABM-2k-100 Prediction	OPENABM-5k-20 Reconstruction	OPENABM-5k-20 Prediction
DYANE	Average	52.2 ± 0.1	51.7 ± 0.1	51.9 ± 0.1	51.9 ± 0.1
	Hadamard	76.4 ± 0.1	72.4 ± 0.2	**90.5 ± 0.3**	77.8 ± 0.2
	Weighted-L1	70.3 ± 0.1	67.4 ± 0.2	78.2 ± 0.7	70.5 ± 0.3
	Weighted-L2	70.3 ± 0.1	67.7 ± 0.1	78.8 ± 0.5	70.9 ± 0.3
	Concat	53.8 ± 0.1	54.6 ± 0.1	52.5 ± 0.1	52.5 ± 0.2
HOSGNS$^{(stat)}$	Average	54.6 ± 0.1	55.4 ± 0.2	55.1 ± 0.2	55.6 ± 0.2
	Hadamard	**91.1 ± 0.1**	**87.0 ± 0.1**	**98.7 ± 0.1**	**86.0 ± 0.1**
	Weighted-L1	69.8 ± 0.1	68.3 ± 0.2	72.7 ± 0.1	69.4 ± 0.1
	Weighted-L2	72.7 ± 0.1	70.7 ± 0.2	76.6 ± 0.1	72.7 ± 0.1
	Concat	56.5 ± 0.1	56.5 ± 0.1	57.4 ± 0.1	57.7 ± 0.1
HOSGNS$^{(dyn)}$	Average	54.0 ± 0.2	54.2 ± 0.2	54.7 ± 0.1	54.3 ± 0.2
	Hadamard	**78.7 ± 0.1**	**79.8 ± 0.2**	82.8 ± 0.3	**82.4 ± 0.2**
	Weighted-L1	71.5 ± 0.3	74.0 ± 0.3	78.5 ± 0.1	77.8 ± 0.2
	Weighted-L2	73.1 ± 0.2	75.6 ± 0.3	80.5 ± 0.1	79.6 ± 0.2
	Concat	57.1 ± 0.1	57.5 ± 0.1	57.5 ± 0.1	57.8 ± 0.1

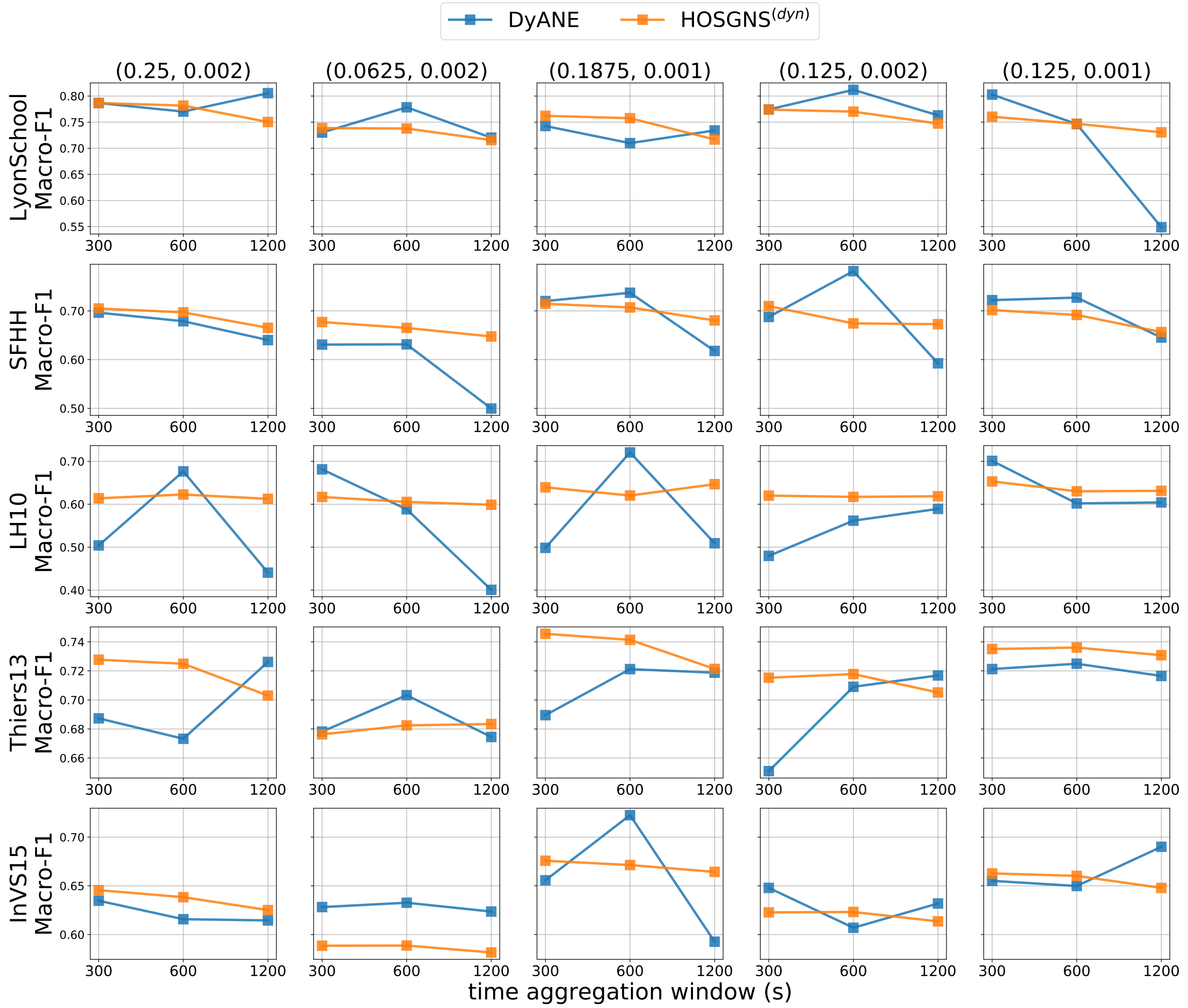

Figure 5: Average macro-F1 scores related to node classification of epidemic states for different SIR processes, varying the time window used to aggregate empirical datasets, with embedding dimension $d = 128$.

performances with analogous low-rank CP decomposition[7]. In displayed tables we show that HOSGNS performs better compared with the corresponding baseline.

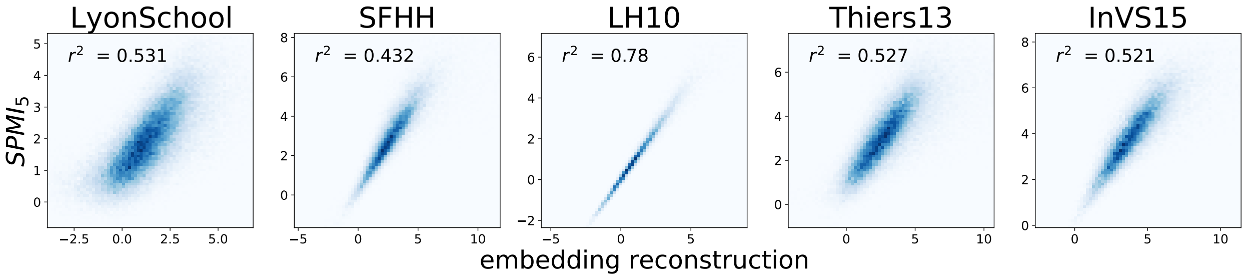

(a) $\text{HOSGNS}^{(stat)}$

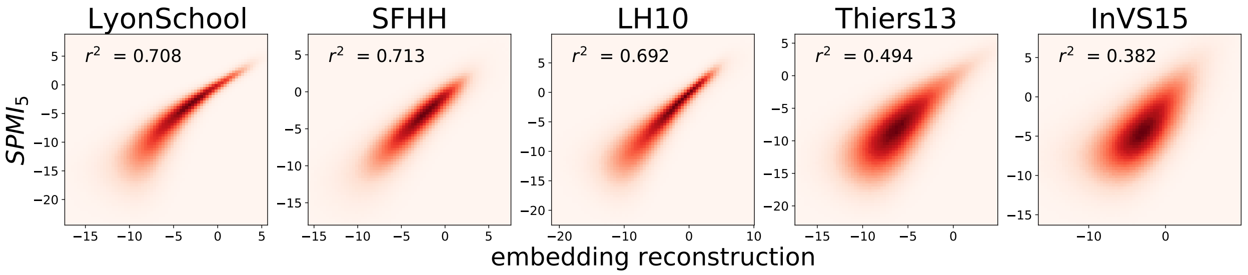

(b) $\text{HOSGNS}^{(dyn)}$

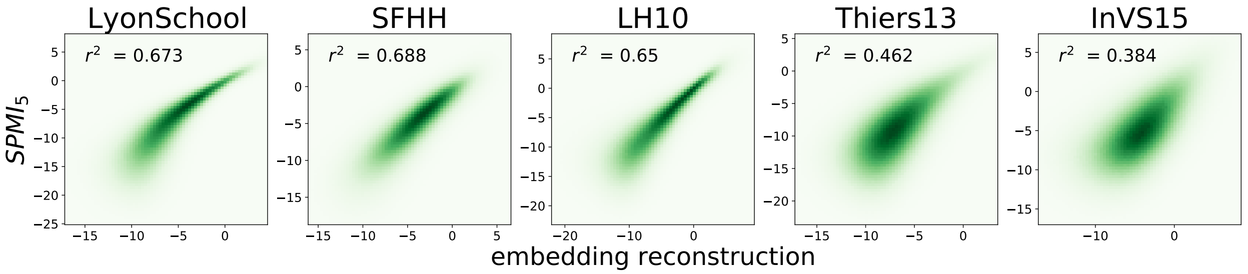

(c) $\text{HOSGNS}^{(stat|dyn)}$

Figure 6: 2D histograms of shifted PMI values $\text{SPMI}_5(i, j, k \dots)$ (whereas are greater than $-\infty$) versus embedding reconstruction from higher-order inner products $[\![\mathbf{w}_i, \mathbf{c}_j, \mathbf{t}_k, \dots]\!]$, with HOSGNS models trained on: (a) $\boldsymbol{\mathcal{P}}^{(stat)}$, (b) $\boldsymbol{\mathcal{P}}^{(dyn)}$ and (c) $\boldsymbol{\mathcal{P}}^{(stat|dyn)}$. The histograms were built by uniformly sampling 10^7 entries from the SPMI_5 tensors.

[7] `https://github.com/tensorly/tensorly`

Table 7: Macro-F1 scores for classification of nodes in epidemic states according to the SIR processe with $(\beta, \mu) = (0.125, 0.001)$. Here HOSGNS variants are compared with tensor factorization baselines, and tested with different operators to produce node-time representations, all with a dimension $d = 64$, used as input to a Logistic Regression. We highlight in bold the best score between HOSGNS and CPTF.

Model	Operator	Dataset LYONSCHOOL	SFHH	LH10	THIERS13	INVS15
3-CPTF	Average	52.2 ± 1.5	58.6 ± 2.4	48.9 ± 1.4	56.6 ± 1.2	51.8 ± 0.7
	Hadamard	32.7 ± 0.6	38.5 ± 2.4	37.9 ± 1.1	31.5 ± 0.4	34.3 ± 0.5
	Weighted-L1	52.1 ± 1.4	59.2 ± 2.4	48.9 ± 2.3	56.4 ± 1.1	50.0 ± 0.9
	Weighted-L2	51.9 ± 1.4	59.2 ± 2.4	44.2 ± 2.2	55.6 ± 1.0	46.0 ± 1.0
	Concat	52.6 ± 1.4	50.5 ± 0.8	45.2 ± 2.1	54.1 ± 0.9	46.6 ± 1.1
HOSGNS$^{(stat)}$	Average	61.2 ± 1.2	65.1 ± 2.3	$\mathbf{53.8 \pm 1.9}$	60.1 ± 0.5	55.6 ± 0.8
	Hadamard	56.1 ± 0.9	$\mathbf{66.1 \pm 2.3}$	48.0 ± 1.0	46.9 ± 0.6	47.9 ± 0.6
	Weighted-L1	56.1 ± 0.6	60.3 ± 2.5	47.0 ± 2.2	53.2 ± 0.9	48.2 ± 0.6
	Weighted-L2	57.0 ± 0.7	59.4 ± 2.6	44.8 ± 1.2	51.8 ± 0.6	46.3 ± 0.8
	Concat	$\mathbf{66.6 \pm 1.2}$	65.9 ± 2.2	52.2 ± 1.9	$\mathbf{63.8 \pm 0.5}$	$\mathbf{58.4 \pm 0.6}$
4-CPTF	Average	70.1 ± 1.1	70.7 ± 2.1	52.5 ± 2.6	62.5 ± 0.8	49.6 ± 1.2
	Hadamard	59.9 ± 0.9	61.6 ± 2.8	46.1 ± 2.6	39.8 ± 0.8	45.2 ± 0.9
	Weighted-L1	70.6 ± 1.1	70.5 ± 2.0	51.2 ± 2.4	63.6 ± 1.0	49.5 ± 1.3
	Weighted-L2	62.3 ± 1.6	66.0 ± 2.4	49.7 ± 2.5	57.9 ± 1.1	44.2 ± 1.0
	Concat	69.2 ± 0.6	67.0 ± 2.3	46.1 ± 2.7	61.4 ± 0.7	48.4 ± 1.0
HOSGNS$^{(dyn)}$	Average	74.1 ± 0.6	71.3 ± 1.9	60.4 ± 2.1	72.9 ± 0.5	65.1 ± 0.6
	Hadamard	$\mathbf{76.0 \pm 0.5}$	$\mathbf{71.5 \pm 1.8}$	$\mathbf{60.4 \pm 1.9}$	$\mathbf{74.3 \pm 0.4}$	65.0 ± 0.6
	Weighted-L1	73.6 ± 0.7	70.5 ± 1.9	58.9 ± 1.7	72.7 ± 0.5	64.2 ± 0.6
	Weighted-L2	73.8 ± 0.5	70.9 ± 1.9	57.0 ± 2.0	72.2 ± 0.6	64.6 ± 0.6
	Concat	73.3 ± 0.6	71.3 ± 1.9	57.3 ± 2.0	72.7 ± 0.5	$\mathbf{65.5 \pm 0.6}$

Table 8: Macro-F1 scores for temporal event reconstruction. Here HOSGNS variants are compared with tensor factorization baselines, and tested with different operators to produce link-time representations, all with a dimension $d = 64$, used as input to a Logistic Regression. We highlight in bold the best score between HOSGNS and baselines.

Model	Operator	Dataset LYONSCHOOL	SFHH	LH10	THIERS13	INVS15
3-CPTF	Average	58.3 ± 0.4	50.7 ± 0.6	48.4 ± 0.9	53.6 ± 0.4	50.5 ± 0.5
	Hadamard	81.7 ± 0.4	55.8 ± 0.9	65.9 ± 1.0	67.0 ± 0.9	50.7 ± 1.2
	Weighted-L1	56.8 ± 0.6	51.8 ± 0.6	49.1 ± 0.6	52.3 ± 0.3	51.5 ± 0.4
	Weighted-L2	56.2 ± 0.4	50.8 ± 0.7	48.3 ± 0.8	53.5 ± 0.3	50.3 ± 0.5
	Concat	61.3 ± 0.4	50.5 ± 0.5	52.9 ± 1.0	54.1 ± 0.4	49.8 ± 0.5
HOSGNS$^{(stat)}$	Average	62.2 ± 0.6	54.6 ± 0.5	55.1 ± 0.7	56.8 ± 0.4	50.2 ± 0.4
	Hadamard	$\mathbf{93.7 \pm 0.2}$	$\mathbf{94.2 \pm 0.2}$	$\mathbf{98.3 \pm 0.2}$	$\mathbf{97.0 \pm 0.1}$	$\mathbf{98.1 \pm 0.1}$
	Weighted-L1	70.6 ± 0.4	61.4 ± 0.4	57.9 ± 0.8	68.7 ± 0.3	62.9 ± 0.5
	Weighted-L2	70.2 ± 0.3	60.9 ± 0.4	57.6 ± 0.6	68.5 ± 0.4	60.0 ± 0.4
	Concat	63.3 ± 0.5	54.5 ± 0.4	52.2 ± 1.0	58.7 ± 0.7	50.5 ± 0.5
4-CPTF	Average	57.8 ± 0.6	50.6 ± 0.5	51.0 ± 0.8	53.2 ± 0.4	49.5 ± 0.6
	Hadamard	71.3 ± 0.6	57.0 ± 0.6	55.8 ± 0.9	45.6 ± 0.7	45.1 ± 0.7
	Weighted-L1	56.5 ± 0.6	50.3 ± 0.7	53.2 ± 0.9	54.3 ± 0.4	50.1 ± 0.4
	Weighted-L2	56.7 ± 0.4	50.9 ± 0.5	49.1 ± 0.7	53.0 ± 0.3	50.2 ± 0.5
	Concat	63.1 ± 0.6	52.5 ± 0.6	50.9 ± 1.1	56.0 ± 0.6	49.6 ± 0.6
HOSGNS$^{(dyn)}$	Average	66.0 ± 0.5	52.6 ± 0.4	52.7 ± 0.9	57.8 ± 0.6	50.4 ± 0.4
	Hadamard	$\mathbf{89.4 \pm 0.2}$	$\mathbf{74.6 \pm 0.4}$	$\mathbf{63.2 \pm 0.6}$	$\mathbf{92.3 \pm 0.2}$	$\mathbf{82.8 \pm 0.4}$
	Weighted-L1	79.6 ± 0.4	60.5 ± 0.4	54.2 ± 0.8	80.9 ± 0.3	63.3 ± 0.5
	Weighted-L2	78.8 ± 0.4	59.1 ± 0.7	54.0 ± 0.8	77.7 ± 0.4	59.3 ± 0.4
	Concat	64.7 ± 0.5	53.9 ± 0.4	50.9 ± 0.9	58.2 ± 0.7	50.9 ± 0.5

Table 9: Macro-F1 scores for missing event prediction. Here HOSGNS variants are compared with tensor factorization baselines, and tested with different operators to produce link-time representations, all with a dimension $d = 64$, used as input to a Logistic Regression. We highlight in bold the best score between HOSGNS and baselines.

Model	Operator	Dataset LYONSCHOOL	SFHH	LH10	THIERS13	INVS15
3-CPTF	Average	57.2 ± 0.6	50.8 ± 1.0	49.0 ± 1.3	53.6 ± 0.5	49.1 ± 0.7
	Hadamard	76.7 ± 0.5	53.4 ± 0.8	56.0 ± 1.2	63.9 ± 0.9	43.0 ± 1.1
	Weighted-L1	56.8 ± 0.5	48.7 ± 0.8	47.9 ± 1.1	53.1 ± 0.6	49.8 ± 0.7
	Weighted-L2	56.4 ± 0.6	45.7 ± 0.9	49.3 ± 1.1	53.0 ± 0.5	48.5 ± 0.7
	Concat	59.2 ± 0.5	53.7 ± 0.8	50.8 ± 1.1	55.5 ± 0.5	50.8 ± 0.7
HOSGNS$^{(stat)}$	Average	61.7 ± 0.5	54.0 ± 0.8	53.7 ± 1.1	57.5 ± 0.7	49.1 ± 0.6
	Hadamard	$\mathbf{85.1 \pm 0.2}$	$\mathbf{56.2 \pm 0.5}$	36.5 ± 0.6	$\mathbf{77.8 \pm 0.3}$	52.8 ± 0.5
	Weighted-L1	67.8 ± 0.6	55.4 ± 0.8	58.1 ± 1.0	62.1 ± 0.7	$\mathbf{55.8 \pm 0.6}$
	Weighted-L2	68.1 ± 0.6	56.2 ± 0.7	$\mathbf{58.2 \pm 1.0}$	63.4 ± 0.6	55.1 ± 0.6
	Concat	61.6 ± 0.6	54.6 ± 0.8	52.3 ± 1.2	59.3 ± 0.6	48.8 ± 0.7
4-CPTF	Average	58.0 ± 0.6	50.7 ± 0.7	50.2 ± 1.4	53.2 ± 0.5	49.0 ± 0.7
	Hadamard	66.8 ± 0.5	49.4 ± 0.8	50.7 ± 1.1	43.1 ± 0.9	36.9 ± 0.6
	Weighted-L1	57.6 ± 0.6	47.3 ± 0.7	50.6 ± 1.2	53.0 ± 0.5	49.0 ± 0.6
	Weighted-L2	56.0 ± 0.6	46.5 ± 0.9	47.5 ± 1.0	53.0 ± 0.6	48.2 ± 0.7
	Concat	60.2 ± 0.6	53.1 ± 0.8	52.5 ± 1.6	55.4 ± 0.6	50.6 ± 0.7
HOSGNS$^{(dyn)}$	Average	65.1 ± 0.6	54.4 ± 0.9	51.9 ± 1.4	59.3 ± 0.7	48.4 ± 0.6
	Hadamard	$\mathbf{89.3 \pm 0.3}$	$\mathbf{72.0 \pm 0.8}$	$\mathbf{62.5 \pm 1.1}$	$\mathbf{91.3 \pm 0.3}$	$\mathbf{78.5 \pm 0.6}$
	Weighted-L1	80.4 ± 0.6	59.7 ± 0.8	53.9 ± 1.3	79.6 ± 0.4	59.9 ± 0.8
	Weighted-L2	79.8 ± 0.5	59.0 ± 0.9	51.9 ± 1.1	78.7 ± 0.4	55.8 ± 0.8
	Concat	63.0 ± 0.6	53.9 ± 0.8	48.4 ± 1.7	60.1 ± 0.8	48.9 ± 0.7

References

[1] Kolda, T.G., Bader, B.W.: Tensor decompositions and applications. SIAM review **51**(3), 455–500 (2009)

[2] Goyal, P., Dollár, P., Girshick, R., Noordhuis, P., Wesolowski, L., Kyrola, A., Tulloch, A., Jia, Y., He, K.: Accurate, large minibatch sgd: Training imagenet in 1 hour. arXiv preprint arXiv:1706.02677 (2017)

[3] Devlin, J., Chang, M.-W., Lee, K., Toutanova, K.: Bert: Pre-training of deep bidirectional transformers for language understanding. arXiv preprint arXiv:1810.04805 (2018)

[4] Kingma, D.P., Ba, J.: Adam: A method for stochastic optimization. arXiv preprint arXiv:1412.6980 (2014)
